# A pan-influenza antibody inhibiting neuraminidase via receptor mimicry

**DOI:** 10.1038/s41586-023-06136-y

**Published:** 2023-05-31

**Authors:** Corey Momont, Ha V. Dang, Fabrizia Zatta, Kevin Hauser, Caihong Wang, Julia di Iulio, Andrea Minola, Nadine Czudnochowski, Anna De Marco, Kaitlin Branch, David Donermeyer, Siddhant Vyas, Alex Chen, Elena Ferri, Barbara Guarino, Abigail E. Powell, Roberto Spreafico, Samantha S. Yim, Dale R. Balce, Istvan Bartha, Marcel Meury, Tristan I. Croll, David M. Belnap, Michael A. Schmid, William Timothy Schaiff, Jessica L. Miller, Elisabetta Cameroni, Amalio Telenti, Herbert W. Virgin, Laura E. Rosen, Lisa A. Purcell, Antonio Lanzavecchia, Gyorgy Snell, Davide Corti, Matteo Samuele Pizzuto

**Affiliations:** 1 Vir Biotechnology, San Francisco, CA USA; 2 Humabs Biomed SA, a subsidiary of Vir Biotechnology, Bellinzona, Switzerland; 3 Vir Biotechnology, St. Louis, MO USA; 4 Cambridge Institute for Medical Research, Department of Haematology, University of Cambridge, Cambridge, UK; 5 School of Biological Sciences, Department of Biochemistry, University of Utah, Salt Lake City, UT USA; 6 Department of Pathology and Immunology, Washington University School of Medicine, St. Louis, MO USA

**Keywords:** Influenza virus, Influenza virus

## Abstract

Rapidly evolving influenza A viruses (IAVs) and influenza B viruses (IBVs) are major causes of recurrent lower respiratory tract infections. Current influenza vaccines elicit antibodies predominantly to the highly variable head region of haemagglutinin and their effectiveness is limited by viral drift^1^ and suboptimal immune responses^2^. Here we describe a neuraminidase-targeting monoclonal antibody, FNI9, that potently inhibits the enzymatic activity of all group 1 and group 2 IAVs, as well as Victoria/2/87-like, Yamagata/16/88-like and ancestral IBVs. FNI9 broadly neutralizes seasonal IAVs and IBVs, including the immune-evading H3N2 strains bearing an N-glycan at position 245, and shows synergistic activity when combined with anti-haemagglutinin stem-directed antibodies. Structural analysis reveals that D107 in the FNI9 heavy chain complementarity-determinant region 3 mimics the interaction of the sialic acid carboxyl group with the three highly conserved arginine residues (R118, R292 and R371) of the neuraminidase catalytic site. FNI9 demonstrates potent prophylactic activity against lethal IAV and IBV infections in mice. The unprecedented breadth and potency of the FNI9 monoclonal antibody supports its development for the prevention of influenza illness by seasonal and pandemic viruses.

## Abstract (web summary)

The neuraminidase-targeting monoclonal antibody FNI9 potently inhibits the enzymatic activity of influenza A and B viruses via receptor mimicry.

## Main

Seasonal influenza viruses represent major causes of severe respiratory tract infections resulting in 300,000–600,000 deaths per year worldwide^3^. IAVs have a vast host tropism that includes different animal species such as swine, bats and various wild birds, which represent the primary reservoir of these viruses. This wide tropism fosters viral evolution and leads to zoonotic infections as well as pandemics^4^. IBVs predominantly circulate in humans and display lower genetic diversity but can cause severe disease especially in children and high-risk individuals^5^.

IAV and IBV infectivity relies on two glycoproteins expressed on the viral envelope that work in concert^6^. Haemagglutinin (HA) binds to the sialic acid (SA) receptor and is pivotal for endosomal membrane fusion, leading to virus uncoating^7^. Neuraminidase (NA) is a receptor-destroying enzyme that cleaves SA, facilitating the release of viral particles from infected cells^8^. NA is also crucial for penetration of the virus through the mucus layer by cleaving decoy receptors that are abundantly present in mucins^9,10^. Owing to their complementary roles, HA and NA activities must be finely balanced to allow viral entry and egress from host cells^11,12^.

Despite the lower abundance on the viral envelope than HA^8,13^, NA represents a key target for neutralizing antibodies. The protective role of anti-NA antibodies has been documented following the 1968 pandemic in which the H3N2 virus carried an NA antigenically related to the previously circulating H2N2 virus^14^. In addition, more recent studies have demonstrated that serum antibodies with NA inhibition (NAI) activity are an independent correlate of protection against IAV infection^15–17^. Besides blocking the interaction of the enzymatic pocket with SA receptors, NA antibodies can also promote Fc-mediated protection in vivo^18^. Although NA neutralizing antibodies can be induced by natural infection, the anti-NA immunity elicited by current influenza vaccines is weak, mainly due to the low abundance and poor stability of the NA antigen, and to the lack of standardization of the amount of NA in approved vaccines^19^.

In view of the antigenic diversity of IAVs and IBVs, the identification of monoclonal antibodies (mAbs) covering both viruses and their variants remains challenging. Several anti-HA stem neutralizing mAbs that recognize the antigenically distant groups 1 and 2 HAs of IAVs have been identified. When tested in a therapeutic setting, these mAbs showed no or only limited efficacy^20,21^. However, one of these mAbs is currently being tested in a phase II prophylactic study^22^. Anti-NA mAbs with broad reactivity may represent a complementary and possibly synergistic approach to anti-HA stem mAbs.

Recent studies described anti-NA neutralizing mAbs isolated following infection or vaccination, showing breadth across several IAVs and IBVs^23–26^. Here we identify a mAb, designated FNI9, which displays an unprecedented breadth and potency against both IAVs and IBVs via receptor mimicry. These findings together with the synergy with HA stem-directed mAbs support the development of FNI9 for the prevention of influenza illness by seasonal and pandemic viruses.

## Isolation of pan-influenza anti-NA mAbs

**Fig. 1:**
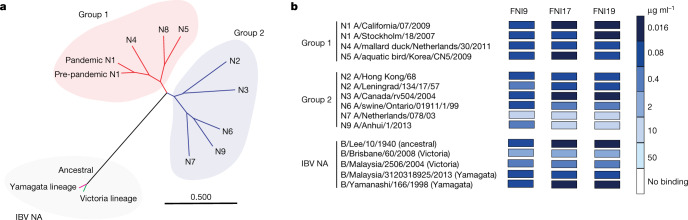
Breadth of FNI9, FNI17 and FNI19 anti-NA mAbs across IAVs and IBVs. **a**, Phylogenetic tree of IAV and IBV NAs constructed using maximum likelihood analysis of amino acid sequences. The scale bar indicates the average number of amino acid substitutions per site. **b**, Heat map of mAb binding to cell-surface-expressed NAs representative of IAV and IBV strains by flow cytometry. Coloured boxes represent the lowest concentration at which mAb binding (expressed as mean fluorescence intensity) was measurable.

**Extended Data Fig. 1:**
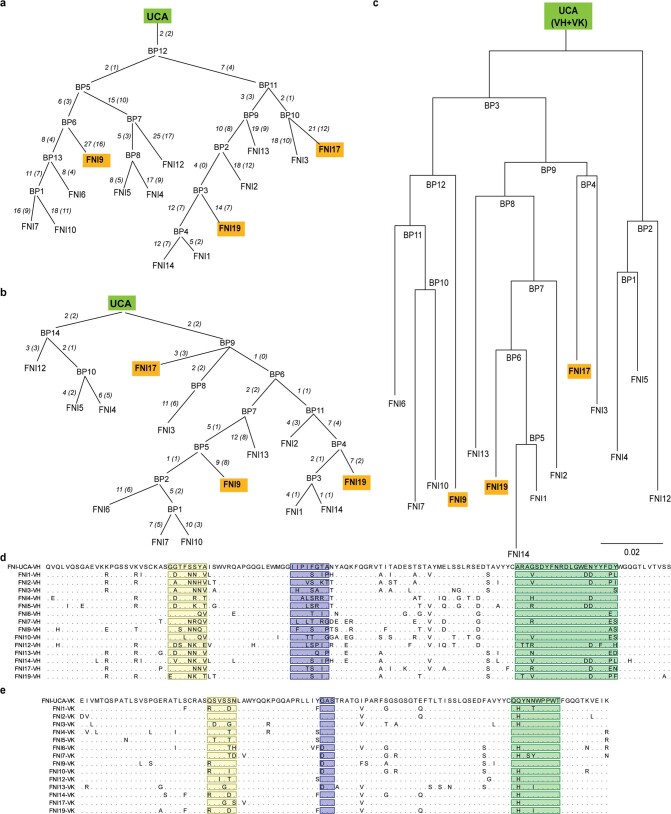
Clonal evolution of FNI mAbs. **a, b**, Graphical representation of the FNI clonal evolution based on the VH (a) and VK (b) generated by the graphic user interface (GUI) AncesTree^53^. Branch lengths are denoted on the lines connecting the branching points with both the total number of nucleotide and amino acid changes, the latter in parentheses. **c**, Phylogenetic tree of the concatenated heavy and light chain sequences generated using raxml-ng^54^ assuming the same single somatic substitution process across both heavy and light chain genes. Each heavy chain sequence was appended to the light chain sequence of the same clone for the purpose of phylogenetic reconstruction under the assumption of a single substitution process. Branch points represent hypothetical bifurcations during the evolution of the antibody sequences. Scale represents the number of amino acid changes per site. **d, e**, Amino acid sequence alignment of FNI clonal family VH (d) and VK (e) with respective unmutated common ancestor (UCA).

Sera from almost 200 healthy individuals were screened for the presence of IgG cross-reacting antibodies to non-human circulating group 1 (N4) and group 2 (N3 and N9) NAs transiently expressed on the surface of mammalian cells (Fig. 1a). One donor, designated ‘I’, with high reactivity against these NA antigens was selected. More than 10 million peripheral blood mononuclear cell-derived IgG^+^ memory B cells from this donor were interrogated using a N1-based baiting strategy (N1 from H5N1 A/Vietnam/1203/2004) and 14,625 cells were sorted and then seeded as single cells in co-culture with mesenchymal stem cells. Culture supernatants were screened for inhibition of N1 sialidase activity by an enzyme-linked lectin assay (ELLA). As ELLA also identifies antibodies that inhibit NA activity by steric hindrance, we further screened the NAI activity of the antibodies in the cell supernatants using the 2′-(4-methylumbelliferyl)-α-D-*N*-acetylneuraminic acid (MUNANA) substrate to select mAbs that directly target the enzymatic pocket of group 1 (N1) and group 2 (N2) NAs. Fourteen mAbs (designated FNI mAbs) were identified and found to belong to a single clonal family diversified by a high load of somatic mutations. These antibodies use *IGHV1-69* and *IGK3-15* genes and are characterized by heavy chain complementarity-determinant region 3 (HCDR3) that is 21 amino acids long (Extended Data Fig. 1a–e and Supplementary Table 1).

**Extended Data Fig. 2:**
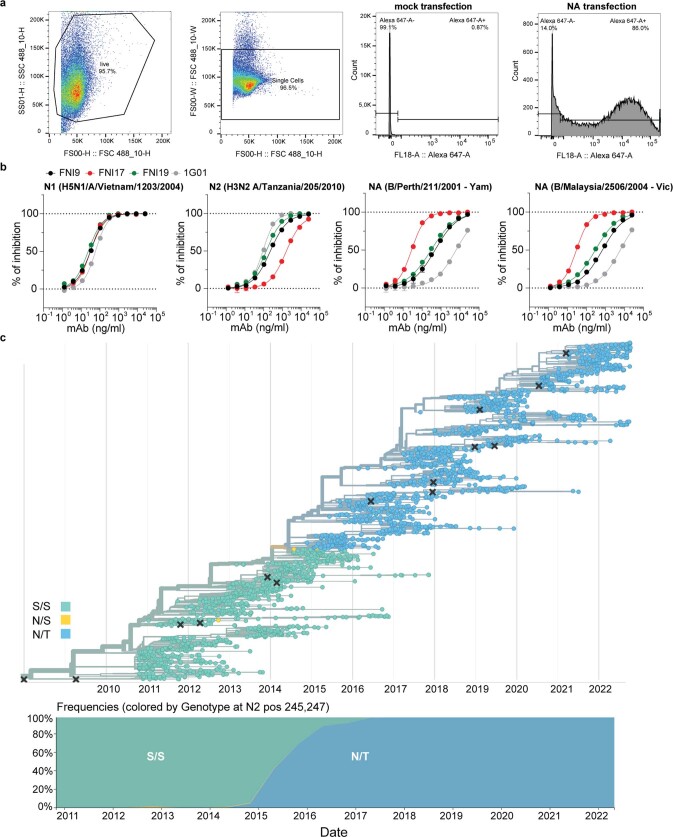
Breadth of FNI mAbs across the Influenza A and B viruses. **a**, FACS gating strategy used to assess binding of FNI and 1G01 mAbs to NA transiently expressed in mammalian cells. **b**, Inhibition of NA enzymatic activity by the anti-NA mAbs against group 1 (N1), group 2 (N2) IAV and B/Victoria/2/87-like (Vic) and B/Yamagata/16/88-like (Yam) IBV NA antigens as measured by MUNANA assay. Data represent n = 1 biologically independent experiment out of two. **c**, Introduction and spreading of the 245 glycosylation motif in H3N2 seasonal viruses. GISAID analysis (2011–2022) of the N2 NAs from seasonal H3N2 strains bearing S245N and S247T mutations, which results in the introduction of the N245 glycosylation site. X represents strains selected for inclusion in the vaccine formulations. Source: Nextstrain (https://nextstrain.org/flu/seasonal/h3n2/na/12y?c=gt-NA_245,247&dmin=2004-10-11). Source Data

Three mAbs—FNI9, FNI17 and FNI19—stood out for their binding to a broad panel of cell-surface-displayed seasonal and animal circulating IAV and IBV NAs (Fig. 1a,b, Extended Data Fig. 2a and Supplementary Table 2). To confirm their broad NAI activity, the three mAbs were tested in a MUNANA assay with N1, N2 and IBV NA antigens (Extended Data Fig. 2b). The previously described broadly neutralizing anti-NA mAb 1G01 (ref. ^24^) was also included as a positive control. FNI9 and FNI19 displayed NAI activity similar to 1G01 to N1 and N2 NAs but stronger inhibition of enzymatic activity to the IBV NAs. FNI17 demonstrated the weakest NAI activity versus N2 antigens but the strongest inhibition of IBV NAs (Extended Data Fig. 2b).

**Fig. 2:**
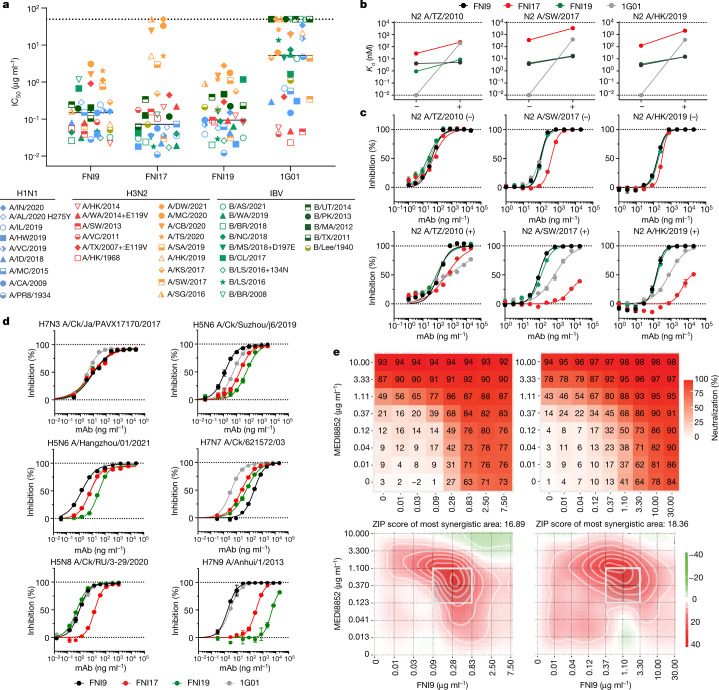
In vitro characterization of FNI9, FNI17 and FNI19 mAbs. **a**, FNI9, FNI17, FNI19 and 1G01 mAbs neutralization half-maximal inhibitory concentration (IC_50_) values against a panel of seasonal IAVs (H1N1 is blue; H3N2 pre-2015 is in red and H3N2 post-2015 is in orange) and IBVs (B/Victoria/2/87-like viruses are in light green, B/Yamagata/16/88-like viruses are in dark green and ancestral is in olive). Full viral strain designations and IC_50_ values are listed in Supplementary Table 3. Geometric mean of *n* = 2 independent experiments for each strain is shown. The black solid line indicates the median IC_50_ for each mAb. Symbols on the dotted line represent strains against which the mAbs did not reach IC_50_ at the concentrations tested in the assay (see Supplementary Table 3). **b**, Binding affinity (*K*_d_) of anti-NA Fab fragments to N2 NA antigens not bearing (−) or bearing (+) glycan at position 245 as measured by SPR. Full details of the antigens tested and *K*_d_ values are reported in Supplementary Table 4. Results represent an average of at least two technical replicates from one independent experiment. Dotted lines represent the limits of detection. **c**, Inhibition of enzymatic activity, as measured by ELLA, exerted by anti-NA mAbs on NA antigens not bearing (−) or bearing (+) N245 glycan. Full details of the antigens used are reported in Supplementary Table 4. **d**, Inhibition of enzymatic activity, as measured by ELLA, exerted by anti-NA mAbs against NA-only based pseudoparticles bearing N3, N6, N7, N8 or N9 representative of zoonotic isolates. Dotted lines in **c** and **d** represent the minimum and maximum percentage of inhibition. Results represent two technical replicates from one independent experiment out of two. Error bars indicate s.d. of technical duplicates. **e**, In vitro neutralization matrixes (top panels) and synergy plots (bottom panels) reporting combination activity of anti-HA stem-directed MEDI8852 and anti-NA FNI9 mAbs to H1N1 A/Puerto Rico/8/34 (left) and H3N2 A/Tasmania/503/2020 (right) viruses. Neutralization matrixes were performed in technical triplicates with one of two independent experiments shown. Source Data

We next assessed FNI9, FNI17 and FNI19 mAbs for their neutralizing activity against a panel of seasonal IAV and IBV strains spanning more than 80 years of NA antigenic evolution (Fig. 2a and Supplementary Table 3). The three FNI mAbs efficiently neutralized all IBVs tested, with FNI17 showing the highest potency in line with the NAI results. Conversely, 1G01 mAb neutralized only a fraction of IBVs tested, consistent with its reported limited coverage of IBV strains^24^. Potent neutralization was observed for all the mAbs to seasonal H1N1 strains (Fig. 2a). Of note, 1G01, but not FNI mAbs, was affected by the oseltamivir-resistant mutation H275Y^27^ (Fig. 2a), which was present in up to 68% of H1N1 seasonal strains pre-2009 H1N1 pandemic^28^. Although all anti-NA mAbs efficiently neutralized the H3N2 strains isolated before 2015, the activity of FNI17 and 1G01 was strongly impaired by the most recent H3N2 isolates (Fig. 2a). Since the 2014/2015 Northern Hemisphere influenza season, a new N-linked glycosylation site at position 245 was introduced on the N2 NA as a result of the S245N and S247T substitutions (Extended Data Fig. 2c). This glycan, which is located at the entry of the NA enzymatic pocket, has been present since then in all seasonal H3N2 viruses, and has been shown to reduce the activity of antibodies targeting the catalytic site^29,30^.

To dissect the effect of the N245 glycan on antibody activity, we produced a panel of three N2 antigens derived from H3N2 strains isolated before and after 2015 together with their counterparts in which the glycosylation site was either introduced or removed (Supplementary Table 4). Surface plasmon resonance (SPR) analysis revealed that FNI17 Fab has weaker affinity than FNI9, FNI19 and 1G01 towards N2 antigens even when the glycan N245 was not present, and glycosylation further decreased its binding (Fig. 2b and Supplementary Table 4). Although the 1G01 Fab displayed the highest binding affinity in the absence of the glycosylation, the presence of the N245 glycan severely dampened 1G01 binding to all N2 antigens tested (Fig. 2b). Despite the N245 glycan being associated with decreased binding to N2, FNI9 and FNI19 retained nanomolar binding affinity (Fig. 2b). Consistent with SPR results, all mAbs showed similar inhibition of N2 enzymatic activity when the N245 glycan was not present, but the introduction of the glycosylation resulted in a marked decrease of NAI activity by FNI17 and 1G01 compared to FNI9 and FNI19 (Fig. 2c). Our results for 1G01 are in line with those recently reported^31^, indicating a decrease in NAI activity by this mAb to N2 bearing the N245 glycan, albeit 1G01 in vivo protection was retained at the single dose tested. Overall, FNI9 and FNI19 mAbs were found to be the most active to contemporary H3N2 strains carrying the N245 glycosylation as well as to oseltamivir-resistant strains.

**Extended Data Fig. 3:**
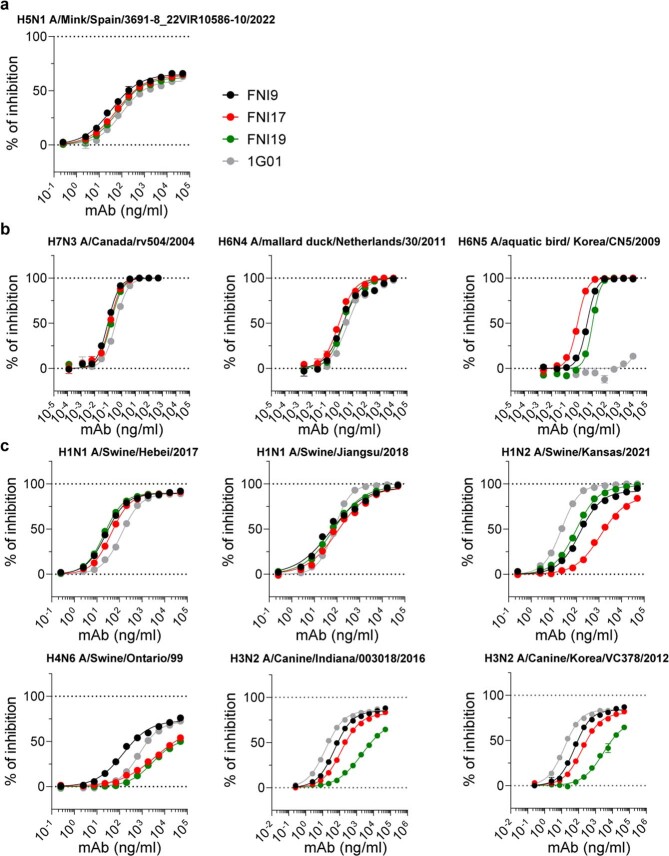
NAI activity of FNI and 1G01 mAbs against pseudoparticles bearing NAs from avian and mammalian influenza A viruses. **a**, Inhibition of sialidase activity of FNI and 1G01 mAbs versus NA-based pseudoparticles bearing N1 from highly pathogenic H5N1 A/Mink/Spain/3691-8_22VIR10586-10/2022 (clade 2.3.4.4) as measure by MUNANA assay. **b**, Inhibition of neuraminidase enzymatic activity, as measured by ELLA, exerted by anti-NA mAbs against NA-based pseudoparticles bearing N3, N4, or N5 representative of enzootic low pathogenic avian influenza A viruses. **c**, Inhibition of sialidase activity of FNI and 1G01 mAbs versus NA-only based pseudoparticles bearing NA representative of enzootic swine or canine influenza A viruses as measured by MUNANA assay. Data from n = 1 biologically independent experiment out of two are shown. Source Data

Next, we assessed the ability of the FNI mAbs to inhibit the enzymatic activity of pseudoparticles bearing NAs representative of enzootic and zoonotic avian and mammalian IAVs. FNI mAbs displayed different inhibitory activity against pseudoparticles bearing NAs from highly pathogenic avian IAVs that were previously reported to have infected humans^32^ or were responsible of the recent outbreak in farmed mammals^33^ (Fig. 2d and Extended Data Fig. 3a). Of note, FNI9 showed the highest NAI activity against N9 from H7N9 among the clonally related antibodies. FNI mAbs displayed comparable NAI activity to pseudoparticles decorated by low pathogenic avian influenza viruses N3, N4 or N5, with the latter not being sensitive to the 1G01 mAb (Extended Data Fig. 3b). Finally, the FNI9 mAb demonstrated broad inhibitory activity across a panel of pseudoparticles presenting NAs from enzootic IAVs circulating in swine and dogs (Extended Data Fig. 3c).

**Extended Data Fig. 4:**
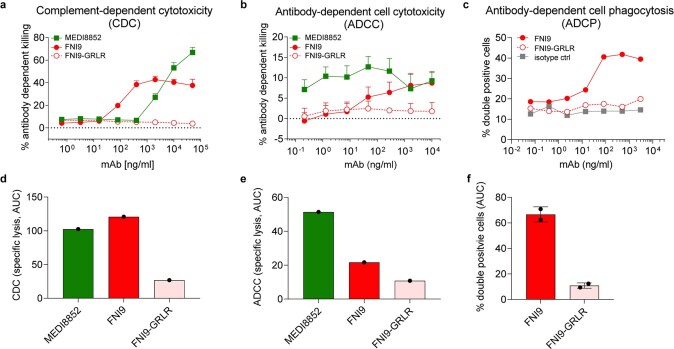
FNI9 mAb mediates activation of CDC, ADCC and ADCP. **a,d**, CDC with a serial dilution of FNI9 (red) and anti-HA MEDI8852 (green) mAbs on MDCK-LN cells infected with H1N1 PR8 in the presence of guinea pig complement. **b,e**, ADCC with FNI9 (red) and anti-HA MEDI8852 (green) mAbs on A549 cells infected with H1N1 PR8 in the presence of freshly isolated human NK cells. **c,f**, ADCP with serial dilution of FNI9 mAb (red) using peripheral blood mononuclear cells (PBMCs as source of monocytes) as effector cells, and PKH67-labelled ExpiCHO cells expressing N2 NA as target cells. The y-axis indicates the percentage of monocytes double positive for CD14 and PKH67. For all assays FNI9-GRLR (red empty circle) is used as Fc-silent negative control and results are shown both as dose-response curves (a-c) and as area under the curve (AUC) (d-f). Results are representative of n = 1 (d, e) or n = 2 (f) biological replicates (black dots). Source Data

Besides inhibiting enzymatic activity and viral replication in vitro, FNI9 induced complement-dependent cell cytotoxicity, mediated the lysis of infected cells by human primary natural killer cells (antibody-dependent cell cytotoxicity) and promoted antibody-dependent cell phagocytosis by monocytes (Extended Data Fig. 4).

The potential interaction of anti-NA mAbs with other influenza virus-targeting antibodies was investigated by combining FNI9 with the broadly neutralizing HA-stem directed mAb MEDI8852 (ref. ^34^). In a checkerboard neutralization assay, these mAbs were found to strongly synergize with a 1:1 anti-HA:anti-NA ratio against both H1N1 A/Puerto Rico/8/34 and H3N2 A/Tasmania/503/2020 (Fig. 2e), suggesting that synergistic activity of broadly neutralizing mAbs could be exploited to increase the breadth and barrier to viral resistance.

Collectively, we identified broadly neutralizing NA-targeting mAbs with unprecedented breadth to seasonal and zoonotic IAVs and IBVs, which can synergize with anti-HA stem-directed mAbs.

## Receptor mimicry by FNI mAbs

**Fig. 3:**
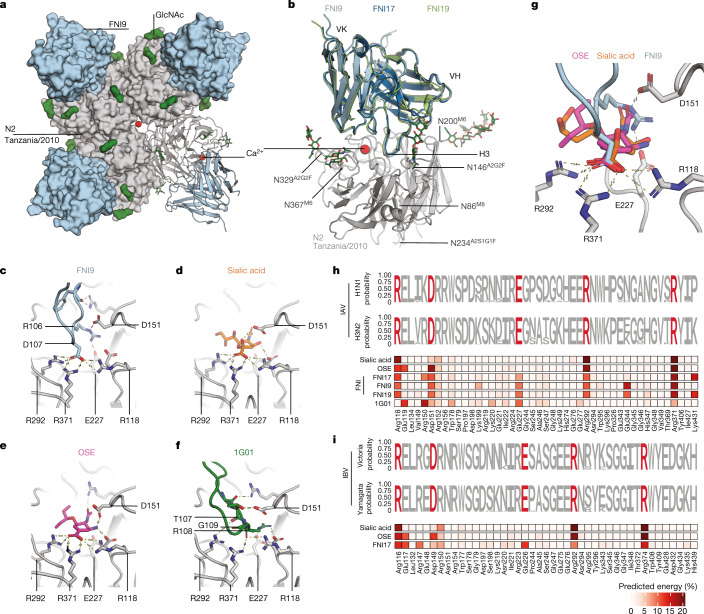
Structure of anti-NA mAbs targeting the SA-binding site. **a**, Topology of the complex formed by FNI9 (light blue) binding to NA (grey). Representative calcium ions at the centre of the tetramer and in the NA–Fab interface are represented as red spheres. Glycans decorating each NA protomer are shown in green (see Extended Data Fig. 7). **b**, Binding of FNI9 (light blue), FNI17 (dark blue) and FNI19 (light green) to the NA (grey) SA-binding pocket. Only the variable domains of the mAbs are shown. VH, variable domain heavy chain; VK, variable domain kappa light chain. **c**, Network of salt bridges and hydrogen bond interactions between R106 and D107 (light blue sticks) in the HCDR3 of FNI9 and R118, D151, E227, R292 and R371 in NA (grey sticks). The dashed lines depict interactions within 3.5 Å. **d**, Network of salt bridges and hydrogen bonds between SA (orange) and NA-binding pocket residues (grey) based on PDB: 4GZQ. The dashed lines are depicted as in **c**. **e**, Network of salt bridges and hydrogen bonds between oseltamivir (OSE; pink) and NA-binding pocket residues (grey) based on PDB: 4GZP. The dashed lines are depicted as in **c**. **f**, Hydrogen bonds between residues T107, R108 and G109 (green) in the HCDR3 of 1G01 (PDB: 6Q23) and the NA-binding pocket residues (grey). The dashed lines are depicted as in **c**. **g**, Overlay of SA, OSE and the HCDR3 of FNI9 illustrates the molecular mimicry of their carboxylates participating in a tridentate salt bridge with R118, R292 and R371. The dashed lines are depicted as in **c**. **h**,**i**, Logo plot amino acid conservation of SA, OSE, FNI9, FNI17, FNI19 and 1G01 epitopes based on available NA sequences from human seasonal H1N1 (*n* = 64,476) and H3N2 (*n* = 91,754) IAVs (**h**) and Victoria/2/87-like (*n* = 23,787) and Yamagata/16/88-like (*n* = 17,769) IBVs (**i**). Key contact residues are shown in red. The binding energies of epitope residues (as a percentage of the total) are shown in the heat map; for the IBV analyses, PDB 1NSC (SA, B/Beijing/1/87) and 4CPY (OSE, B/Lyon/CHU/15.216/2011) were used. Source Data

To elucidate the structural basis for the FNI9, FNI17 and FNI19 broad neutralizing activity, we determined the cryo-electron microscopy structures of their Fabs in complex with NA from H3N2 A/Tanzania/205/2010 at 2.9 Å, 2.3 Å and 3.1 Å resolutions, respectively (Supplementary Table 5). The overall topology of the FNI9–NA complex adopts a C4 symmetry, with each NA protomer bound to a Fab (Fig. 3a). FNI9, FNI17 and FNI19 all adopt the same binding mode (Fig. 3b), inserting the HCDR3 into the enzymatic pocket where residues R106 and D107 establish multivalent salt bridges with the NA active site residues R118, D151, R292 and R371 (ref. ^35^) (Fig. 3c).

**Extended Data Fig. 5:**
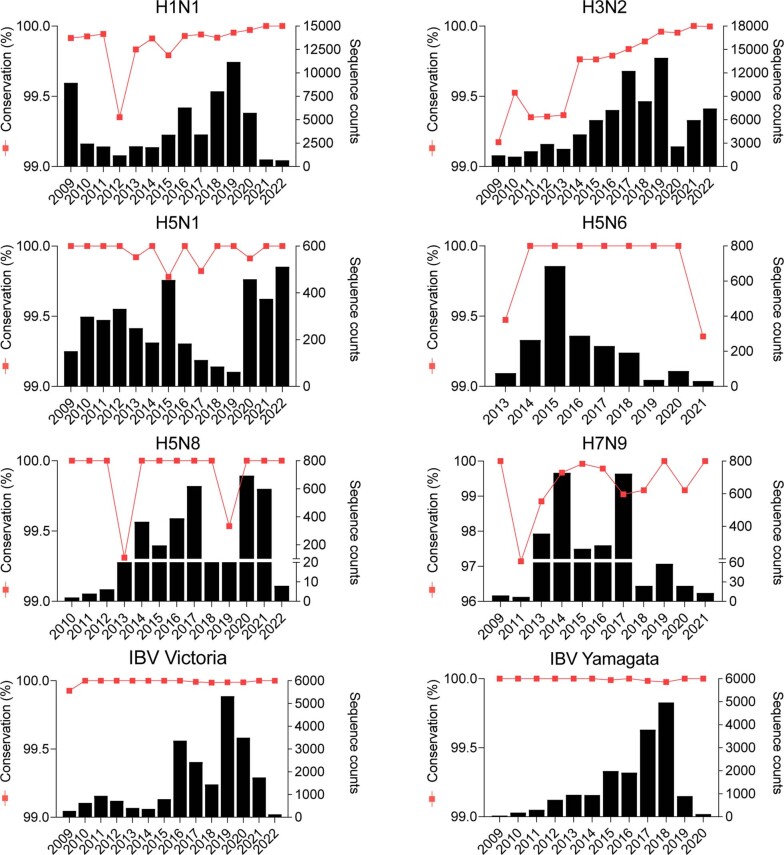
Conservation analysis of FNI mAbs key contact residues on the NA of IAVs and IBVs. Mean conservation percentage (2009–2022) of key NA contacting residues (R118, D151, E227, R292, and R371) per year (red line). The number of sequences analysed per year is shown (black bars). Sequences retrieved for H1N1, H3N2 and IBV isolates are of human origin, while those for H5N1, H7N9, H5N8 and H5N6 viruses are of both human and animal origin. Sequences were retrieved from GISAID database. IAV sequences conservation was measured against A/New York/392/2004 reference strain. IBV sequences conservation was measured against B/Yamagata/16/88 reference strain. Source Data

**Extended Data Fig. 6:**
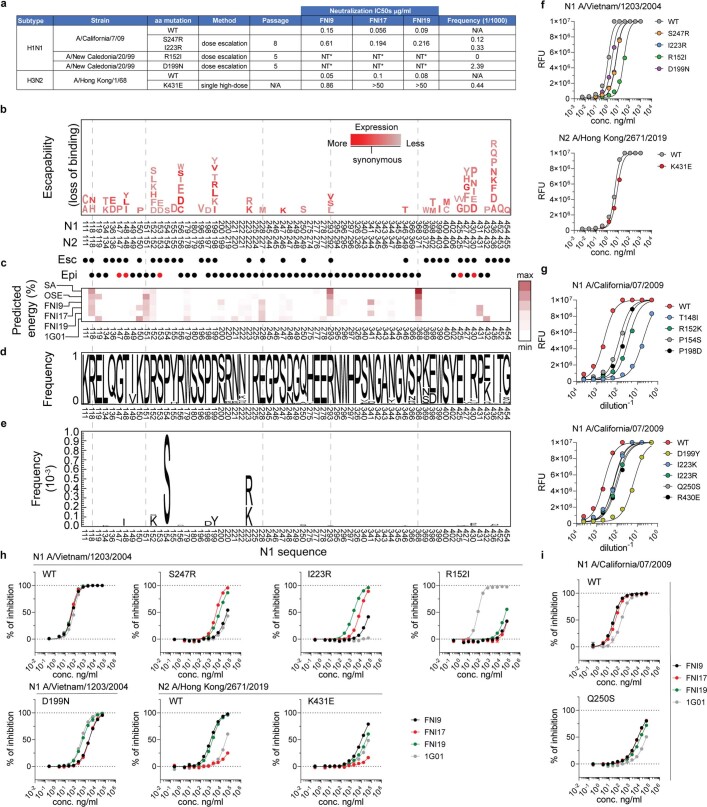
Escape mutants, frequency and fitness cost. **a**, Summary table showing amino acid substitutions identified in resistance studies with H1N1 and H3N2 viruses, the number of virus passages that were required to observe the mutation, the neutralization IC50s against the mutants and the frequency of the mutation in all IAVs deposited in GISAID between 2000–2022. NT*: not tested as R152I and D199N mutants were not rescued following virus propagation in vitro. Note: IC50s observed with S247R/I223R and K431E mutants are in the range of the overall IC50s observed for FNI9 with seasonal IAVs and IBVs (see Fig. 2a). **b**, Mutations reducing FNI9 binding to N1 from H1N1 A/California/07/2009 identified by DMS. Only amino acid substitutions that do not result in strong reduction of expression are reported in the red colour scale (darker red, more expression; lighter red, less expression in comparison to synonymous mutations). N1 numbering is used on the x-axis along with N2 numbering below. N2 positions identified as epitope residues by static structural analysis are highlighted with black dots (‘Epi’) and additional positions identified by dynamic analysis (MD) are highlighted with red dots. Potential escape mutations flagged by DMS (top row of black dots, ‘Esc’) not identified as epitope residues have low frequencies in GISAID (see below). **c**, Static epitope analysis of SA, oseltamivir (OSE), FNI9, FNI17, FNI19, and 1G01 shown as in Fig. 3h, where lighter red is a weaker interaction and darker red is a stronger interaction. **d**, Logo plot of N1 sequences in GISAID shows the frequencies of all residues at each position across all species (n = 79,316). N1 numbering is used on the x-axis and the y-axis extends from a cumulative frequency of zero to one. The potential escape mutants in (b) are too infrequent to be seen in (d). **e**, Frequency of only the DMS-identified potential escapes appearing in GISAID for N1 across all species and all time windows (n = 79,316) shown as a logo plot. N1 numbering is used on the x-axis and the y-axis extends from a cumulative frequency of zero to 10^−3^. **f**, Enzymatic activity, as measured by MUNANA, of N1 antigen from H5N1 A/Vietnam/1203/2004 or N2 antigen from H3N2 A/Hong Kong/2671/2019 bearing amino acid substitutions identified via resistance studies (a) in comparison to the sialidase activity of the wild type NAs. A detailed list of the mutations and NA antigens in which they were introduced is reported in Supplemetary Table 4. **g**, Enzymatic activity, as measured by MUNANA, of pseudoparticles bearing N1 from H1N1 A/California/07/09 with amino acid substitutions retrieved from DMS (b-e) in comparison to pseudoparticles presenting the parental NA. **h,i**, Inhibition of neuraminidase enzymatic activity by FNI and 1G01 mAbs, as measured by MUNANA, of a panel of NA bearing mutations identified through resistance studies (h) or DMS (i). Data for n = 1 biologically independent experiment are shown. Source Data

These four NA active site residues also form the primary interactions with SA, suggesting that the broad neutralizing activity of FNI mAbs is the result of receptor molecular mimicry (Fig. 3d,g), similar to the binding mode of oseltamivir (Fig. 3e,g). By contrast, 1G01 interactions within the enzymatic pocket are distinct from those observed for the SA receptor, oseltamivir and the FNI mAbs (Fig. 3f). Of note, both 1G01 and FNI mAbs also interact with E227, which contributes to form the NA active site framework (Fig. 3c,f). Owing to the functional constraints associated with the SA receptor interactions, the key contact residues of the FNI mAbs are highly conserved across IAV and IBV NAs (Fig. 3h,i and Extended Data Fig. 5). Moreover, in vitro resistance studies with live virus as well as deep mutational scanning via an NA library expressed in mammalian cells revealed a very limited number of NA mutations capable of reducing, but not abrogating, the binding or activity of FNI mAbs (Extended Data Fig. 6 and Supplementary Table 4). Of note, most of these mutations were only retrieved after several rounds of viral passages, are associated with a decrease in the NA sialidase activity and are extremely rare in human and animal isolates, suggesting a fitness cost for the corresponding virus mutants and a high barrier to resistance for this class of anti-NA antibodies (Extended Data Fig. 6).

**Extended Data Fig. 7:**
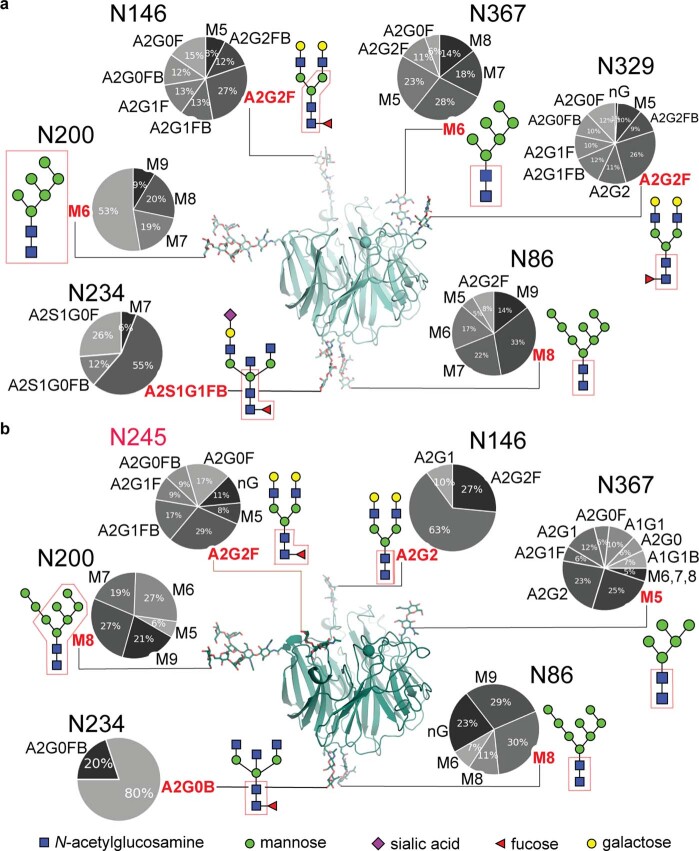
Glycan profiling of neuraminidase with peptide mapping LC-MS. **a**, Glycan profile of N2 A/Tanzania/2010 with pie charts depicting the percent distributions of glycans. The most abundant glycan (red font) is illustrated; the monosaccharide units resolved by cryoEM are outlined in red. The most abundant glycan at each position in N2 was modelled onto cryoEM structures to prepare the MD simulations (see Methods for details). **b**, Glycan profile of N2 A/Hong Kong/2019 depicted as in (a). Oxford glycan notation is used; nG, no glycan. Source Data

**Extended Data Fig. 8:**
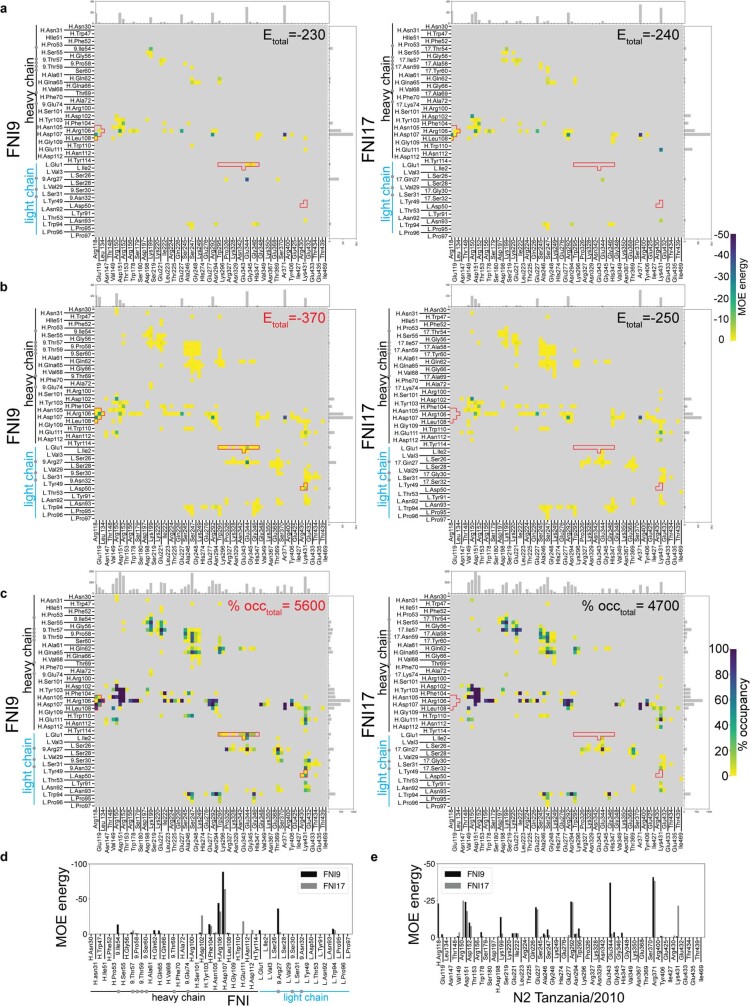
Detailed structural analysis of FNI9 and FNI17 mAbs binding to NA. **a**, Static epitope analysis of FNI9:NA (left) and FNI17:NA (right) on N2 A/Tanzania/2010 depicted as a heatmap whose color scale is MOE kcal/mol energy; x-axis delineates epitope residues; and y-axis delineates paratope residues. FNI9 and FNI17 sequence differences are specified by grey dots along the y-axes and total binding energies are reported. **b**, Dynamic epitope analysis of FNI9:NA (left) and FNI17:NA (right) on N2 A/Tanzania/2010 depicted as in (a) using energies derived from 11.5 µs MD simulations. Red squares depict epitope-paratope residue contacts present in the FNI9:NA complex that are absent in the FNI17:NA complex. Dynamic total binding energies are reported. **c**, Percent occupancy of epitope-paratope interactions (within 5 Å) of FNI9:NA (left) and FNI17:NA (right) on N2 A/Tanzania/2010 in the 11.5 µs MD simulations. Percentage of total occupancy is reported. **d**, Comparison of FNI9 and FNI17 paratopes using dynamic analysis; FNI9 and FNI17 sequence differences are specified by grey dots along the x-axis. **e**, Comparison of FNI9 and FNI17 epitopes using dynamic analysis; NA residues depicted along the x-axis. Source Data

To obtain a more detailed understanding of the binding epitopes of FNI mAbs, molecular dynamics (MD) simulations of FNI–NA complexes were performed using the complete glycans revealed by peptide mapping liquid chromatography–mass spectrometry (Extended Data Fig. 7). Although the analysis of the static structures shows similar binding energies for the FNI9–NA and FNI17–NA complexes (Extended Data Fig. 8a), the MD-derived total dynamic binding energy predicts that FNI9 is a stronger binder (−370 MOE (molecular operating environment) kcal per mol) than FNI17 (−250 MOE kcal per mol) for NA from the H3N2 A/Tanzania/205/2010 strain (Extended Data Fig. 8b,c), in line with binding affinity measurements (Fig. 2b and Supplementary Table 4). The interactions are balanced across more residues in the FNI9–NA complex than in the FNI17–NA complex both in the epitope (Extended Data Fig. 8d) and in the paratope (Extended Data Fig. 8e), providing a possible mechanistic explanation for the superior binding of FNI9.

**Fig. 4:**
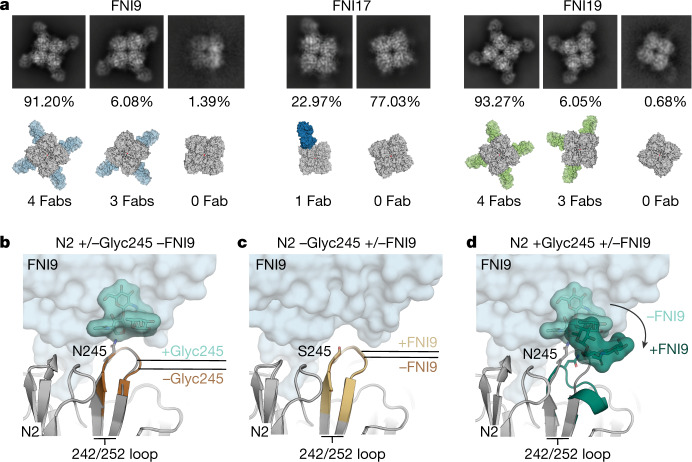
FNI mAbs induce a conformational change in the 242/252 loop when N245 is glycosylated. **a**, 2D and 3D classifications and percentages of classes of FNI9–NA (N2 A/Hong Kong/2019) showing four, three and zero Fabs bound to the tetramer (the condition with a single Fab bound is not shown: 1.33%), FNI17–NA (N2 A/Tanzania/2010 with S245N and S247T) showing one and zero Fabs bound to the NA tetramer, and FNI19–NA (N2 A/Hong Kong/2019) with zero, three and four Fabs bound to the NA tetramer. **b**, N245 glycosylated (+Glyc245; grey) and non-glycosylated (−Glyc245; brown) NAs reveal very similar conformations of the 242/252 loop in the unbound state. Overlay of the FNI9 Fab (light blue, translucent surface) indicates the steric hindrance with the N245 glycan (light green surface), which would occur without a rearrangement of the 242/252 loop and the glycan. **c**, Overlays of N2 A/Tanzania/2010 NA (−Glyc245) with (+FNI9, gold) and without (−FNI9, brown) Fab bound reveal that the NAs adopt indistinguishable conformations; the FNI9 Fab is shown as a light blue surface. **d**, Overlays of N245-glycosylated NA structures with (+FNI9, dark green) and without (−FNI9, grey) Fab bound illustrate the influence of the Fab on inducing a conformational change in the 242/252 loop. Although the peptide mapping liquid chromatography–mass spectrometry in Extended Data Fig. 7 revealed that position 245 bears an A2G2F glycan, only the two GlcNAcs and fucose resolved by cryo-electron microscopy are shown in the figure. Source Data

We next investigated the structural basis for the reduced binding affinities to N2 antigens bearing the N245 glycan. Two-dimensional classification of the cryo-electron microscopy data showed the presence of N2 tetramers carrying the N245 glycan saturated with three or four FNI9 Fabs (6.08% with three FNI9 Fabs and 91.20% with four FNI9 Fabs) or FNI19 Fabs (6.05% with three FNI19 Fabs and 93.27% with four FNI19 Fabs), whereas only zero or one FNI17 Fabs (77.03% with zero FNI17 Fabs and 22.97% with one FNI17 Fab) were found to occupy each tetramer (Fig. 4a). These data are consistent with the lower binding affinity and neutralizing activity of FNI17 than FNI9 and FNI19 versus the N2 antigens and viruses bearing the N245 glycan (Fig. 2a,b).

Structural analysis revealed that the conformation of the 242/252 loop, which contains the N_245_AT_247_ glycosylation motif, is not influenced by the presence or absence of the N245 glycan (Fig. 4b). In N2 NA structures without the glycan, there is no clash with the antibody and the 242/252 loop adopts virtually indistinguishable conformations in both bound and unbound states (Fig. 4c). By contrast, following binding of the FNI9 antibody, both the 242/252 loop and the N245 glycan undergo a dramatic conformational change to avoid a clash between the Fab and the glycan (Fig. 4d). The energy required for the induced fit of loop 242/252 could underlie the differences in affinity and neutralizing activity of the FNI mAbs against the recent N2 NAs.

## Prophylactic activity of the FNI9 mAb

**Fig. 5:**
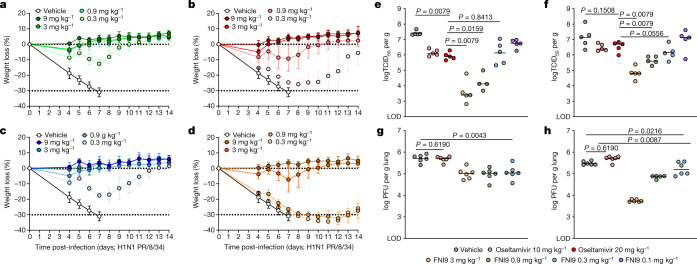
FNI9 mAb protects mice from seasonal IAV and IBV lethal challenges. **a**–**d**, Percentage of body weight loss of BALB/c mice (*n* = 6 mice per group) prophylactically administered with murinized anti-NA FNI9 mAb (**a**), anti-HA stem-directed MEDI8852 mAb (**b**) and the same mAbs bearing the N297Q Fc mutation (**c**,**d**) 24 h before lethal infection with H1N1 A/Puerto Rico/8/34. Doses are reported in different colours and the average body weight loss for each dose group is shown. Error bars represent standard deviations. The 0% dotted line indicates baseline body weight loss; the −30% dotted line indicates body weight loss % for euthanasia based on lead veterinarian assessment as described in the ethical statement. **e**–**h**, Replicating virus titres in the lungs of BALB/c mice measured 4 days post-infection with H1N1 A/Puerto Rico/8/34 (**e**), H3N2 A/Singapore/INFIMH-16-0019/2016 (**f**) (*n* = 5 mice per group), B/Victoria/504/2000 (Yamagato linage) (**g**) and B/Brisbane/60/2008 (Victoria lineage) (**h**) (*n* = 6 mice per group) following prophylactic administration of FNI9 at 3, 0.9, 0.3 mg kg^−1^ (**e**–**h**) and 0.1 mg kg^−1^ (**e**,**f**). Each panel presents data derived from *n* = 1 independent experiment. Two-tailed Mann–Whitney test was used for statistical analysis of significance. LOD, limit of detection; TCID, tissue culture infectious dose. Source Data

**Extended Data Fig. 9:**
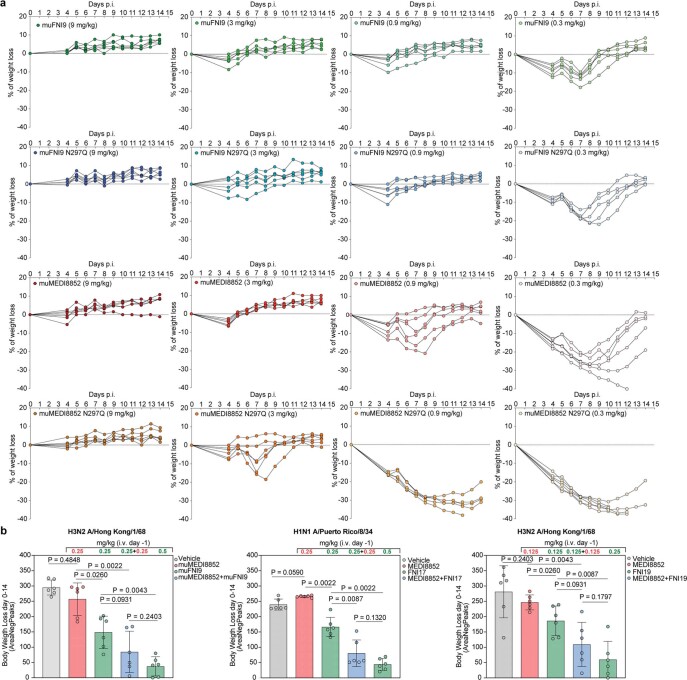
Contribution of effector functions and combination studies in vivo. **a**, Body weight loss of BALB/c mice (n = 6 mice/group) related to Fig. 5a–d. Body weight loss percentage for single BALB/c mice prophylactically administered with muFNI9, muFNI9 N297Q, muMEDI8852, and muMEDI8852 N297Q mAbs at 9, 3, 0.9, and 0.3 mg/kg and infected with a lethal dose of H1N1 A/Puerto Rico/8/34. **b**, Area of negative peaks of the body weigh loss measured for the BALB/c mice (n = 6 mice/group) prophylactically administered with murinized MEDI8852 (muMEDI8852), murinized FNI9 (muFNI9), MEDI8852, FNI17, FNI9 and the combination of the anti-HA and anti-NA mAbs in a 1:1 ratio against H1N1 A/Puerto Rico/8/34 or H3N2 A/Hong Kong/1/1968 as indicated in the graphs. The area of the negative peaks is defined as the area between the body weight loss line of each animal over 14 days and the 0% body weight loss baseline. Data from n = 1 independent experiment for each anti-NA mAb are presented. Two-tailed Mann–Whitney test was used for statistical analysis of significance. Errors bars indicate S.D. Source Data

To assess the in vivo activity of FNI9 in a mouse model, we produced a murinized IgG2a form of this mAb (muFNI9) and a N297Q mutant (muFNI9(N297Q)) that lacks FcRs and complement C1q binding^36^. For comparison, we also produced the same murinized versions of MEDI8852, a broadly IAV neutralizing anti-HA stem antibody^34^. The four mAbs were prophylactically administered via intravenous injection to BALB/c mice 24 h before lethal challenge with H1N1 A/Puerto Rico/8/34. Mice receiving muFNI9 were completely protected from loss of body weight down to 0.9 mg kg^−1^ and displayed limited loss of body weight at 0.3 mg kg^−1^, with all the animals recovering throughout 14 days (Fig. 5a and Extended Data Fig. 9a). Administration of muMEDI8852 conferred full protection from body weight loss down to 3 mg kg^−1^; whereas moderate-to-significant body weight loss was observed at 0.9 and 0.3 mg kg^−1^, respectively (Fig. 5b and Extended Data Fig. 9a). When the same antibodies were tested with the N297Q mutation, muFNI9(N297Q) displayed protection comparable with that of the parental antibody at all the doses tested (Fig. 5c and Extended Data Fig. 9a). By contrast, muMEDI8852 bearing N297Q showed a marked decreased activity at limiting doses (0.9 and 0.3 mg kg^−1^), consistent with previous findings on the role of effector function in protection by anti-HA stem-directed antibodies^37^ (Fig. 5d and Extended Data Fig. 9a). Overall, these data indicate that FNI9 has strong prophylactic activity against lethal infection with H1N1 A/Puerto Rico/8/34, which does not appear to be dependent on Fc-mediated effector functions at the doses tested.

Next, we observed that mice prophylactically administered with an ineffective dose of MEDI8852 combined with FNI mAbs in a 1:1 ratio were overall better protected from morbidity than those receiving only the same amount of FNI mAbs contained in the mix, suggesting that the activity of anti-HA stem antibodies can benefit from the association with NA-inhibiting antibodies (Extended Data Fig. 9b).

Given the similarity in the mechanism of action, we compared the prophylactic activity of a single injection of FNI9 with daily administration of oseltamivir in BALB/c mice lethally challenged with a panel of IAVs and IBVs (Fig. 5e–h). Daily administration of oseltamivir at 10 mg kg^−1^ (ref. ^38^) reduced viral titre by approximately 1 log against IAVs (H1N1 A/Puerto Rico/8/34 or H3N2 A/Singapore/INFIMH-16-0019/2016) and no further viral titre reduction was observed by doubling the dose (20 mg kg^−1^) of the small molecule. Conversely, FNI9 at 3 and 0.9 mg kg^−1^ reduced lung viral titres by 4 and 3 logs after challenge with H1N1 A/Puerto Rico/8/34 and by 3 and 2 logs after challenge with H3N2 A/Singapore/INFIMH-16-0019/2016 that carries the N245 glycan (Fig. 5e,f). A viral lung titre reduction comparable with the one provided by oseltamivir was obtained with both IAVs when FNI9 was administered at 0.3 mg kg^−1^ (Fig. 5e,f).

**Extended Data Fig. 10:**
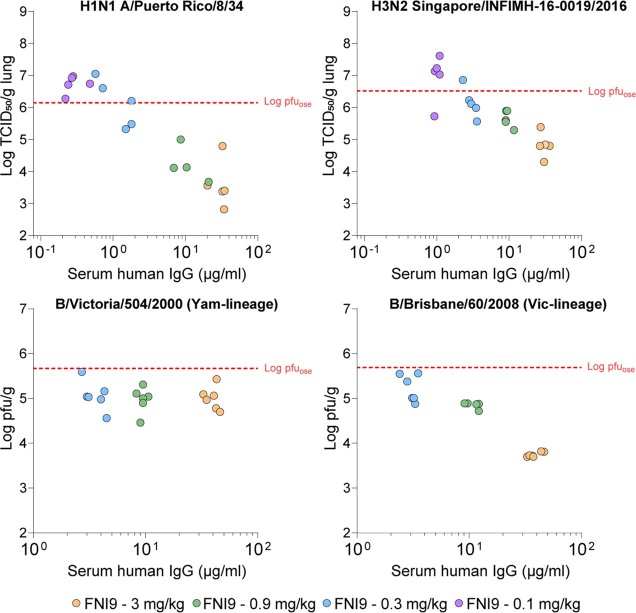
Correlation between mAb concentration at Day 0 and virus lung titres related to Fig. 5e–h. Replicating virus titres in the lungs of BALB/c mice at 4 days after challenge plotted against serum mAb concentrations measured 2 h before infection with H1N1 A/Puerto Rico/8/34, H3N2 A/Singapore/2016, B/Victoria/504/2000, or B/Brisbane/60/2008 viruses. Dotted red lines represent viral lung titre reductions produced by oseltamivir. Details on the mAb quantification are reported in the corresponding material and method section. Source Data

In line with the lower in vitro potency and clinical efficacy against IBVs than IAVs^39,40^, oseltamivir did not show significant activity to IBVs B/Victoria/504/2000 (B/Yamagata/16/88-like virus) and B/Brisbane/60/2008 (B/Victoria/2/87-like virus). Conversely, administration of FNI9 significantly reduced viral lung titres to B/Victoria/504/2000 and B/Brisbane/60/2008 down to 0.3 mg kg^−1^ (Fig. 5g,h). Of note, viral replication in the lungs inversely correlated with the serum mAb concentration measured 2 h before infection (Extended Data Fig. 10).

Collectively, these results indicate that a single administration of FNI9 mAb at 0.3 mg kg^−1^ is highly efficacious in preventing replication of both IAVs and IBVs in the lungs of BALB/c mice.

## Discussion

The effectiveness of current influenza vaccines is often suboptimal due to mismatch with circulating seasonal strains, immunological imprinting and limited immunogenicity, especially in elderly and immunocompromised individuals^2,41^. Previous studies have shown that NA-directed antiviral drugs are effective in prophylaxis^42,43^, albeit their use in this setting is limited by their short half-life and potential selection of escape mutants. Furthermore, anti-NA serum antibodies have been shown to independently correlate with protection from IAV infection^15,44^. In comparison with other previously described anti-NA mAbs^23,24^, the FNI9 mAb presented in this study displays larger breadth and higher potency across seasonal IAVs and IBVs, including contemporary H3N2 strains. The SA receptor molecular mimicry exploited by the FNI9 mAb might provide an additional layer of protection from viral escapes, as the key interacting residues in the epitope are evolutionarily conserved in human and animal IAVs and IBVs and most of the NA mutations retrieved from in vitro resistance studies are associated with a fitness cost for the virus. Of note, molecular mimicry of the SA receptor was also observed for anti-HA mAbs^45^, which displayed limited breadth. The features of the FNI9 mAb combined with half-life extension through Fc modifications^46^ support its development as a single dose per season prophylaxis against influenza.

The synergistic activity observed when FNI9 is combined with the MEDI8852 mAb is consistent with previous reports on the synergy between oseltamivir and anti-HA stem mAbs^34^. In addition, other studies have shown that anti-NA mAbs can increase the Fc-mediated protection by anti-HA stem mAbs^47,48^. We therefore speculate that FNI9 efficacy could be further increased by synergy with endogenous anti-HA stem antibodies elicited by previous infections or vaccinations.

Collectively our findings support the notion that NA is an important target for influenza prophylaxis and justify ongoing efforts to produce recombinant stabilized NA immunogens^49,50^ to elicit broadly reactive NAI antibodies, thus contributing to the development of a universal influenza vaccine.

## Methods

### Cells and viruses

Cell lines were obtained from the American Type Culture Collection (ATCC) (HEK293T/17, Madin-Darby Canine Kidney (MDCK), MDCK-London (MDCK-LN) and A549), or Thermo Fisher Scientific (ExpiCHO-S and Expi293F cells). Expi293F and ExpiCHO-S cells were maintained in Expi293 Expression Medium (Gibco) and ExpiCHO Expression Medium (Gibco), respectively. FreeStyle 293-F cells (Thermo Fisher) used in the DMS experiments were cultured in pre-transduction FreeStyle™ 293 Expression Medium (Thermo Fisher, 12338018) or post-transduction media consisting of DMEM + 10% FBS +1% HEPES + 1% Pen/Strep, with 1.5 μg/mL puromycin for selection. All cell lines used in this study were routinely tested for mycoplasma and found to be mycoplasma-free. Wild-type influenza strains were obtained from the International Reagent Resource (CDC) and the Centre for Biological Reference Materials (NIBSC). Propagation of viral stocks was performed as previously described^51^. In brief, MDCK-LN cells (FR-58, International Reagent Resource) were seeded at 6 × 10^6^ cells in a T75 flask in growth medium (DMEM (11995040, Gibco), 10% FBS (97068-085, VWR), 0.01 M HEPES (15630-080, Gibco), 100 U ml^−1^ penicillin–100 µg ml^−1^ streptomycin (15140-122, Gibco)) and infected the next day at a multiplicity of infection (MOI) of 0.1 in infection medium. Cells were washed twice with PBS before infection. Virus was diluted in infection medium (DMEM, 0.1% BSA, 0.01 M HEPES, 100 U ml^−1^ penicillin–100 µg ml^−1^ streptomycin) and added to cells for 1 h at 37 °C. After absorption, virus inoculum was removed, cells were washed once with PBS, and 10 ml of infection medium containing 2 µg ml^−1^ of l-1-tosylamido-2-phenylethyl chloromethyl ketone (TPCK)-treated trypsin (100 mg; LS003740, Worthington Biochemical) was added to the flasks. The infected cells were incubated at 37 °C for IAV and 35 °C for IBV in a tissue culture incubator for 24 h. Infection medium were then harvested, spun at 2,000*g* for 5 min, and supernatant was collected, aliquoted and stored at −80 °C. Virus titres were determined by focus forming units (FFU) per millilitre, as described below.

### Sample donors

Samples from healthy individuals were obtained from Swiss blood donation centres. Blood donors agreed, by means of a written inform consent form, that their blood or certain components of it could be used anonymously for research purposes. Anonymous blood samples were banked and used for research at VIR Bellinzona in the context of study protocols approved by the local Institutional Review Boards (Canton Ticino Ethics Committee, Switzerland).

### B cell isolation and stimulation

Memory B cells from the selected donors were isolated from cryopreserved peripheral blood mononuclear cells (PBMCs) as follow: CD19^+^ B cells were enriched from PBMCs by staining with phycoerythrin (PE)-Cy7-labelled anti-human CD19 (BD Biosciences) on ice for 30 min, followed by staining with anti-PE beads (Miltenyi Biotec) on ice for 20 min and, finally, positive selected on MACS-separation LS columns (Miltenyi Biotec). NA1 from IAV (N1-IAV) reactive memory IgG^+^ B cells were isolated by staining with a biotinylated recombinant N1 (H5N1 IAV-NA; VIVA Biotech) in complex with streptavidin-AF647 (Life Technologies), anti-human IgA PE (Southern Biotech), anti-human IgM PE (BioLegend) and anti-human IgD PE (BD Bioscience) on ice for 45 min and by fluorescence-activated cell sorting (FACS) on a FACSAria (BD Biosciences). Cells were resuspended in IMDM with 10% FBS in the presence of 2.5 μg ml^−1^ CpG 2006, 500 U ml^−1^ IL-2, 5 ng ml^−1^ IL-6 (BD Pharmingen), 50 ng ml^−1^ IL-10 (ImmunoTools) and 10 ng ml^−1^ IL-21 (ImmunoTools) and seeded under clonal conditions on a monolayer of mesenchymal stem cells in 384-well microtitre plates (Corning). After 8–10 days, the supernatants were screened for their NAI activity in a high-throughput ELLA. The B cell supernatants that were having NAI activity were re-tested in a secondary screening against N1, N2 and N9 (VIVA Biotech) in a NAI assay MUNANA (NI-MUNANA). Cross-reactive memory B cells were lysed, and the variable regions of the heavy and light chain were cloned.

### Sequence analysis of mAbs

Complementary DNA (cDNA) was synthesized from selected B cell culture and both the heavy and the light chain variable region (VH and VL) sequences were retrieved by PCR as previously described^52^. Using the database IMGT (http://www.imgt.org), the VH and VL gene family and the number of somatic mutations were determined by analysing the homology of the VH and VL sequences to known human V, D and J genes. Unmutated common ancestor (UCA) sequences of the VH and VL were constructed using IMGT/V-QUEST.

The DNA sequences of the VH and VL regions of 14 clonally related mAbs (FNI1–FNI20) were aligned using the online multiple sequence alignment program Clustal Omega (https://www.ebi.ac.uk/Tools/msa/clustalo/). Subsequently, the UCA sequence was generated using the UA inference application, and the generation and analysis of the phylogenetic tree were performed with dnamI software and AncesTree software^53^, respectively. A phylogenetic tree of the concatenated heavy and light chain sequences was generated assuming the same single somatic substitution process across both genes. Each heavy chain sequence was appended to the light chain sequence of the same clone for the purpose of phylogenetic reconstruction under the assumption of a single substitution process. A phylogenetic tree and ancestral states were inferred using raxml-ng with the Blosum62 amino acid substitution model^54^.

### Production of recombinant mAbs

The VH and VL regions were cloned into IgG1 and IgΚ expression vectors^52^ and expressed recombinantly by transient transfection of ExpiCHO cells (Thermo Fisher Scientific) using the ExpiFectamine CHO transfection kit (Thermo Fisher Scientific). After 8 days, cell culture supernatants were purified by protein A chromatography on ÄKTA Xpress Fast Protein Liquid Chromatography (Cytiva) using HiTrap Protein A columns (Cytiva) followed by buffer exchange to histidine buffer using HiPrep 26/10 desalting columns (Cytiva). The purified antibodies were quantified using the MAbPac protein A column (Thermo Fisher Scientific). The FNI17 and FNI19 Fabs were recombinantly produced by transient transfection of ExpiCHO using the same conditions reported for the full IgG antibodies. The FNI9 Fab was produced in HEK293 suspension cells. Purification was performed using CaptureSelect IgG-CH1 resin (5943462005, Thermo Scientific) followed by buffer exchange into PBS. The 1G01 Fab was obtained by fragmentation of the corresponding IgGs using the FabALACTICA Fab kit (A2-AFK-025, Genovis) according to the manufacturer’s protocol.

### Transient expression of NA proteins on Expi cells

IAV and IBV NA protein coding sequences were cloned into the phCMV1 expression plasmid (Genlantis) under the control of the human CMV promoter. Immediately before transfection, ExpiCHO-S or Expi293F cells were seeded at 6 × 10^6^ cells per millilitre or 3 × 10^6^ cells per millilitre, respectively, in a volume of 5 ml in a 50-ml bioreactor. Cells were transfected with 5 µg NA plasmids using the ExpiFectamine CHO or ExpiFectamine 293 Transfection Kit (Thermo Fisher Scientific), following the protocol provided with the transfection kit. Transfected cells were then incubated at 37 °C at 8% CO_2_ with an orbital shaking speed of 250 rpm (orbital diameter of 25 mm) for 48 h.

### Flow cytometry analysis

Selection of cross-reactive plasma against group 1 and group 2 IAV NAs and the evaluation of the breadth of the best FNI mAbs were performed by binding to NA proteins expressed on the cell surface by flow cytometry. NA expressing ExpiCHO-S or Expi293F cells were harvested, washed twice in FACS buffer (PBS supplemented with 2% FBS and 2 mM of ethylenediaminetetraacetic acid), counted and distributed into 96-well U-bottom plates (Corning). The sera or mAbs were serially diluted and incubated with the cells for 45 min on ice and then washed in FACS buffer. Alexa Fluor 647-labelled goat anti-human IgG secondary antibody (Jackson Immunoresearch) was added at 1 µg ml^−1^ to the cells, following 20 min of incubation on ice. Cells were then washed with FACS buffer and analysed on a ZE5 Flow Cytometer (Bio-Rad). Data were normalized to the NA-positive cell fractions and analysed with FlowJo software.

### Pseudoparticles production

NA-only based particles (NA-VLPs) were produced by transfecting HEK293T/17 cells in 56.7-cm^2^ cell culture dishes (NUNC). Cells were co-transfected using X-tremeGENE HP (Roche) with 1 µg of NA-expressing plasmid and with 8 µg of a complementing viral-genome reporter vector, pNL4-3.Luc+.E-R+. A 1:3 DNA:X-tremeGENE HP ratio was used and the transfection was performed following the manufacturer’s protocol. Transfected cells were incubated for 72 h, then the supernatant was harvested and stored at −80 °C into aliquots. The pseudoparticles used in this study were generated with NAs from H1N1 A/swine Hebei/0116/2017, H1N2 A/swine/Kansas/A02246977/2021, H7N3 A/Ck/Ja/PAVX17170/2017, H7N3 A/Canada/rv504/2004, H6N4 A/mallard duck/Netherlands/30/2011, H6N5 A/aquatic bird/Korea/CN5/2009, H4N6 A/swine Ontario/01911-1/99, H5N6 A/Hangzhou/01/2021, H5N6 A/Ck/Suzhou/j6/2019, H7N7 A/Ck/621572/03, H5N8 A/Ck/Russia/3-29/2020 and H7N9 A/Anhui/1/2013.

### Production of recombinant NA proteins

NA proteins containing an N-terminal IgK light chain secretion sequence, a 6×His-Tag, AviTag, a tetrabrachion tetramerization domain and a thrombin or tobacco etch virus (TEV) protease site were expressed in Expi293F cells at 37 °C and 8% CO_2_. Cell culture supernatant was collected 4 days post-transfection and proteins were purified using Nickel-NTA agarose (Qiagen). Proteins were buffer exchanged into 50 mM Tris-HCl, 150 mM NaCl and 10 mM CaCl_2_ (pH 8.0) by dialysis or size-exclusion chromatography using a Superdex 200 10/300 GL Increase or Superdex 200 (16/600) (Cytiva) column. NA from A/Tanzania/205/2010, NA B/Perth/211/2001 and NA B/Malaysia/2506/2004 were buffer exchanged into PBS (136.8 mM NaCl, 2.5 mM KCl, 0.8 mM Na_2_HPO_4_, 1.47 mM KH_2_PO_4_, 0.9 mM CaCl_2_ and 0.5 mM MgCl_2_, pH 7.4) by dialysis.

### ELLA

384-Well (NUNC) or 96-well half-area ELISA (Corning) plates were coated overnight at 4 °C with 25 µg ml^−1^ of fetuin. Plates were blocked with a 1% w/v solution of BSA (Sigma-Aldrich) in PBS containing Ca^2+^Mg^2+^ (Gibco) and incubated for 1 h at room temperature. In the meantime, mAbs were serially diluted and mixed with a fixed amount of recombinant NA proteins or NA-VLPs and the mix was transferred to fetuin-coated plates, previously washed. After an overnight incubation at 37 °C, the plates were washed and 1 µg ml^−1^ lectin from peanut-agglutinin (Sigma-Aldrich) labelled with horseradish peroxidase was added and incubated at room temperature for 1 h. After further washing, SureBlue Reserve TMB 1-Component (KPL) was added, and plates were read at 450 nm after blocking the reaction with 1% hydrochloric acid solution.

### MUNANA

mAbs were serially diluted and mixed with a fixed amount of recombinant NA proteins in 96-well black plates (Greiner) or 384-well black plates (TTP Labtech) and incubated for 30 min at 37 °C. Subsequently, the MUNANA substrate (Sigma Aldrich) was added at a final concentration of 16.7 µM. The plates were incubated for 2.5–5 h at 37 °C, then the reaction was stopped with MUNANA stop solution (0.2 M glycine/50% Et-OH, pH 10.7) and the NA activity was measured by fluorescence using Cytation5 (excitation at 365 nm and emission at 445 nm).

### SPR assays to measure binding of Avi-tagged NA proteins by Fabs

Measurements were performed using a Biacore T200 instrument. A CM5 chip with covalently immobilized anti-Avi tag polyclonal antibody (A00674-40, GenScript) was used for surface capture of NA proteins bearing the Avi tag (GLNDIFEAQKIEWHE). Running buffer was filtered with HEPES-buffered saline with added P-20 and calcium chloride (HBS P+ CaCl_2_: 10 mM HEPES (4-(2-hydroxyethyl)-1-piperazineethanesulfonic acid) pH 7.5, 150 mM NaCl, 0.005% w/v P-20 and 10 mM CaCl_2_). The regeneration condition was two injections of 75 mM phosphoric acid for 1 min each. Measurements were performed at 25 °C. Experiments were performed with a fourfold dilution series of monomeric Fabs (800, 200, 50 and 12.5 nM) and were run as single-cycle kinetics.

Data were double-reference subtracted and fit to a binding model using Biacore Insight software. The 1:1 binding model with a constant *R*_max_ was used to estimate the kinetic parameters. For each ligand (NA protein), a constant *R*_max_, which is the maximum of all *R*_max_ values for all Fab analytes from the default 1:1 binding analysis, was applied, under the assumption that all Fabs have the same *R*_max_ for a specific NA protein ligand. The consistent capture level of each ligand over all cycles (within 8%) justifies this assumption. The experiment was performed twice with two technical replicates for each ligand (NA protein). *K*_d_ values were reported as the average of two replicates. For measurements in which the dissociation constant *K*_d_ is slower than the detection range of the instrument (*K*_d_ < 10^−6^ s^−1^), the binding affinity (*K*_d_) is denoted as less than 0.01 nM.

### Virus titration and in vitro neutralization assays

Virus titres were determined by FFU assay on MDCK-LN cells, using immunofluorescence staining. MDCK-LN cells were seeded into flat-bottom tissue culture 96-well plates (3904, Corning) at 25,000 cells per well and cultured overnight at 37 °C in growth medium. Twenty-four hours later, fivefold dilutions of viral stocks were prepared in infection medium containing 0.5 µg ml^−1^ TPCK-treated trypsin, with each dilution tested in quadruplicate. Cells were washed with DMEM medium and then infected with 50 µl of diluted virus. After absorption at 37 °C for 30 min, inoculum was removed, cells were washed once in DMEM medium and 100 µl infection medium with 2.4% colloidal cellulose overlay (435244, Sigma) and 0.5 µg ml^−1^ TPCK-treated trypsin was added to each well. After 24 h of incubation at 37 °C for IAV and 35 °C for IBV, cells were fixed with 4% paraformaldehyde (AA233689L, Fisher) for 30 min at room temperature, washed three times with wash/perm buffer (0.5% Triton X-100 in PBS) to remove residual paraformaldehyde, then the cells were permeabilized with 100 µl wash/perm buffer for 30 min. Cells were incubated for 1 h with 50 µl anti-influenza A or anti-influenza B nucleoprotein antibody (ab128193 or ab20711, Abcam) at 1:1,000 and 0.5% BSA in wash/perm buffer followed by three washes with wash/perm buffer. The cells were then incubated with 50 µl goat anti-mouse IgG Alexa Fluor Plus 647 (A-32728, Invitrogen) at 1:1,000 with 2 µg ml^−1^ Hoechst dye (10 mg ml^−1^; H3570, Invitrogen) in wash/perm buffer for 1 h and then three additional washes with wash/perm buffer. Plates were imaged on a S6 Universal M2 Plate Imager and resulting FFU titres were calculated by counting wells that contained 10–20 distinct foci.

Microneutralization was evaluated in a multicycle assay by adapting a protocol previously described^55^. In brief, MDCK-LN cells were seeded into flat-bottom tissue culture 96-well plates at 25,000 cells per well and cultured overnight at 37 °C in growth medium. Twenty-four hours later, virus stocks were diluted in infection medium containing 0.5 µg ml^−1^ TPCK-treated trypsin for a final concentration of 70 FFU per well, cells were washed once with DMEM medium, then 50 µl of diluted virus was added to the cells and incubated at 37 °C for 30 min. A nine-point 1:5 serial dilution of mAb was prepared in infection medium containing 0.5 µg ml^−1^ TPCK-treated trypsin, with each dilution tested in triplicate (final range: 50,000–0.128 ng ml^−1^). Diluted antibody was mixed 1:1 with colloidal cellulose overlay (final concentration of 2.4%) resuspended in infection medium containing 0.5 µg ml^−1^ TPCK-treated trypsin, then infected cells were washed once with DMEM medium and 100 µl antibody with cellulose overlay was added to the wells. Cells were incubated for 24 h at 37 °C for IAV, 35 °C for IBV B/Victoria/2/87-like viruses and 35 °C for 48 h for IBV B/Yamagata/16/88-like viruses in a tissue culture incubator. Viral replication was measured following the same procedure as described above for the virus titration assay. Plates were imaged on a Cytation5 plate reader. Whole-well images were acquired (12 images at ×4 magnification per well) and nucleocapsid-positive cells were counted using the manufacturer’s software. To control for background, the mean count of positive cells in uninfected wells was calculated and subtracted from all data points. All positive cell counts were normalized to the no-antibody treatment control. Data were analysed and graphed using GraphPad Prism software (v9.0.0). The IC_50_ values were calculated using a non-linear regression model (variable slope model, four parameters) of log(inhibitor) versus response and the IC_50_ values were interpolated from the curve at *y* = 50. The geometric mean of two independent experiments in which the antibody was tested in triplicate was calculated in Excel.

To confirm the actual TCID_50_ of the input virus used in the neutralization assay for each viral strain, a TCID_50_ viral assay was carried out in parallel. For this assay, a twofold serial dilution of input virus in 50 μl was added to duplicate wells containing MDCK-LN cells and incubated in parallel to the microneutralization assay at 37 °C for 30 min. Cells were washed once with DMEM, and 100 µl infection medium containing 0.5 µg ml^−1^ TPCK-treated trypsin was added to the cells and incubated with the neutralization plates. Viral replication was measured following the same procedure as described above for the virus titration assay. Infected wells were observed with the Cytation5 and resulting titres were calculated using the Reed–Muench method^56^. The target viral input for each experiment was 100 TCID_50_ per well.

### Assessment of synergy

Cell plating and virus infections were carried out using the in vitro virus neutralization procedure described above with all infection medium containing 0.5 µg ml^−1^ TPCK-treated trypsin. To assess the synergy between the two monoclonal antibodies, a seven-point 1:3 serial dilution of MEDI8852 was prepared in infection medium (final concentration: 10–0.01372 µg ml^−1^). Subsequently, a nine-point 1:3 serial dilution of FNI9 was prepared in infection medium (final concentration: 7.5–0.001143 µg ml^−1^) and mixed at 1:1 ratio with MEDI8852 in 2-ml deep-well plates creating a drug combo checkerboard matrix. The diluted drug combination was then mixed 1:1 with a colloidal cellulose overlay (final concentration of 2.4%). The infected cells were washed once with DMEM and 100 µl of antibody–drug combination with cellulose overlay was added to the wells, with each condition tested in triplicate. The plates were incubated at 37 °C for 24 h in a tissue culture incubator. Viral replication was measured following the same procedure as described above in the virus titration assay and plates were imaged on the Cytation5 plate reader. Neutralization synergy was calculated by normalizing the signal of the treatment wells to no-antibody virus control wells and no-virus cell control wells and the values were imported to the web-based software SynergyFinder. The zero interaction potency statistical model was utilized to calculate total synergy scores, with values greater than 10 indicating likely synergy between the two drugs. Two independent experiments were performed with three technical replicates for each virus.

### In vitro resistance studies

Antibody-resistant mutants were isolated by screening the viral population in bulk or by serial passaging the virus in the presence of an escalating dose of antibody. For the former approach, MDCK cells were plated into 96-well plates (CON3596, Corning) at 30,000 cells per well in 100 μl of culture medium (MEM + GlutaMAX (41090-028, Thermo Fisher) + 10% FBS Hyclone (SH30070.03, VWR) + 50 U ml^−1^ penicillin–50 ng ml^−1^ streptomycin (4-01F00-H, Bioconcept) + 100 µg ml^−1^ kanamycin (15160047, Life Technologies) + 1% NEAA (5-13K00-H, Bioconcept) + 1% sodium pyruvate (11360039, Life Technologies) + 0.05 mM β-mercaptoethanol (5-69F00-E, Bioconcept)) and cultured overnight at 37 °C at 5% CO_2_. The day after, A/Hong Kong/1/1968 virus input was pre-incubated with 100 μg ml^−1^ of FNI19 antibody for 30 min at 37 °C. The virus–antibody mix was then limiting diluted in infection medium (MEM supplemented with 1 μg ml^−1^ TCPK-trypsin (LS003750, Bioconcept) and 10 μg ml^−1^ kanamycin) to infect MDCK cells at MOI of 0.001 in 100 μl, after two washing steps with 200 μl per well of PBS (D8537-500ML, Sigma-Aldrich). Three hours later, 100 μl per well of infection medium containing 100 μg ml^−1^ FNI19 was added, and cells were incubated for a further 72 h. After the incubation time, a 20 mM MUNANA substrate (69587-5MG, Sigma) solution was prepared in MUNANA buffer (32.5 mM MES and 4 mM CaCl_2_, pH 6.5) and 50 μl per well dispensed into 96-well plate to be incubated with an equal amount of assay culture supernatant for 1 h at 37 °C. Of stop solution (0.2 M glycine and 50% Et-OH, pH 10.7), 100 μl per well was added and fluorescence at 445 nm was measured with a Cytation5 (Biotek-Agilent). Fluorescence threshold was set to 5× the signal of not infected cells and supernatants above that cut-off were processed for NA sequencing. Viral genomic RNA was extracted from 140 μl of culture supernatant using the Qiagen QIAmp Viral RNA kit (52904, Qiagen) according to manufacturer’s instruction. cDNA was synthetized using the SuperScript III RT kit (18080044, Life Technologies) with the primer Uni12 (5′-AGC RAA AGC AGG-3′). The gene encoding NA was amplified by PCR using the Q5 HotStart DNA polymerase kit (M0493L, Bioconcept) with a specific primer forward: 5′-caattggctctgtctctct-3′ and reverse: 5′-atgaaattgatgttcgccc-3′. PCR product was purified from 1.5% agarose gel and sequenced with the three different primers: 5′-caattggctctgtctctct-3′, 5′-gaagagccgatactagaa-3′ and 5′-ttctagtatcggctcttc-3′. Sequence alignment with A/Hong Kong/1/1968 NA was performed with CLC Main Workbench 22 software.

For the serial passaging resistance experiment, MDCK-LN cells were seeded in 24-well plates at 120,000 cells per well and cultured overnight at 37 °C in growth medium (DMEM, 10% FBS, 0.01 M HEPES and 100 U ml^−1^ penicillin–100 µg ml^−1^ streptomycin). Twenty-four hours later, H1N1 A/California/07/2009 or A/New Caledonia/20/99 virus stock and FNI9 were diluted and mixed 1:1 in infection medium containing 0.5 µg ml^−1^ TPCK-treated trypsin for a final concentration of 1,000 PFU per well virus and 0.5× IC_50_ of FNI9 (47.35 ng ml^−1^). The virus–antibody mixture was placed at 37 °C and precomplexed for 30 min. Cell and virus control wells were included for cytophatic effects (CPE) comparators. The cells were washed twice in DMEM and then 100 µl of the virus–antibody complexed mixture, virus and cell controls were added to the wells for 1 h at 37 °C. After virus adsorption, the cells were washed twice in DMEM, then 500 µl of diluted antibody or infection medium containing 0.5 µg ml^−1^ TPCK-treated trypsin was added back to their respective wells. The plates were incubated at 37 °C and the virus was collected once there was over 50% CPE in the virus–antibody well. The virus supernatant was frozen in 120 µl aliquots at −80 °C, one aliquot was used for the next resistance passage, one aliquot was added to 360 µl of DNA/RNA Shield for RNA extraction, and one aliquot was saved for additional propagations. For passage 2, the process was repeated with the original 0.5× and a 1× IC_50_ concentration of FNI9, with the highest concentration of antibody showing CPE being selected for the next passage. For each additional passage, the concentration from the selected well was repeated along with a 2× concentration. For the CA09 study, there were eight passages of virus and a final concentration of 64× the IC_50_ of FNI9 (3,200 ng ml^−1^).

For identification of genomic mutations, viral genomic RNA was extracted using the Zymo QuickRNA Viral Kit (R1034) according to the manufacturer’s instructions and the RNA concentration was measured using the Qubit RNA BR Assay Kit (Q10210, Invitrogen). cDNA was synthesized using the SuperScript III One-Step RT–PCR system with Platinum Taq DNA polymerase (12574018, Invitrogen) using the MBTuni-12 (5′-ACGCGTGATCAGCAAAAGCAGG-3′) and MBTuni-13 (5′-ACGCGTGATCAGTAGAAACAAGG-3′) primers, and the PCR fragments were purified using AMPure XP magnetic beads (A63880, Beckman Coulter). Next, the amplicons were fragmented using the NEBNext Ultra II FS DNA Library Prep Kit for Illumina (E6177L, NEB), then adaptors and indexes were added using NEBNext Multiplex Oligos for Illumina (7335L, NEB). DNA samples were purified using NEBNext Sample purification beads and then quantified using the Qubit DNA HS Assay kit (Q32854, Invitrogen), and verified using a D500 tape station (G2964AA, Agilent). Finally, the samples were diluted and pooled, then sequenced using a MiSEQ analyser with the MiSeq Reagent Kit v3 (MS-102-3003) and PhiX Control v3 (FC-110-3001). The NA mutations I223R and S247N first appeared in passages 6 and 7, respectively, and each reached 50% of the total next-generation sequencing (NGS) reads by passage 8.

### Measurement of Fc-effector functions

To evaluate complement-dependent cytotoxicity (CDC assay), MDCK-LN cells were infected with A/California/07/2009 (H1N1) at an MOI of 6. After 18 h at 37 °C with 5% CO_2_, cells were detached with trypsin, washed and dispensed into flat-bottom 384-well plates. Antibodies were serially diluted in AIM-V medium, mixed with infected target cells and incubated for 10 min at room temperature. After the incubation, guinea pig low tox complement (Cedarlane) previously diluted 1:5 with AIM-V medium was added to each well. Control wells containing target cells and complement with 50 µl of 2% Triton X-100 to measure maximal lysis or target cells and complement only to evaluate spontaneous lysis were also included. Cell death was quantified after 3 h of incubation at 37 °C with 5% CO_2_, by measuring lactate dehydrogenase (LDH) release using the LDH detection kit (11644793001, Roche) according to the manufacturer’s instructions. Using a kinetic protocol, the absorbance at 490 nm and 650 nm was measured once a minute for 8 min, and the percent specific lysis was determined.

Antibody-dependent cell cytotoxicity was performed on A549 cells infected with A/Puerto Rico/8/34 (H1N1) at an MOI of 6. After 18 h at 37 °C with 5% CO_2_, infected target cells were detached with trypsin, washed and dispended into round-bottom 384-well plates (7.5 × 10^3^ per well in AIM-V). The serial dilutions for antibodies were prepared in AIM-V, added onto cells, and incubated for 10 min at room temperature. Human natural killer (NK) cells were isolated from fresh blood using the MACSxpress WB NK cell isolation kit, human (130-127-695, Miltenyi Biotec) and following the manufacturer’s instructions. After the incubation, NK cells were added at a cell density of 4.5 × 10^4^ per well, to reach an effector to target ratio of 6:1. Control wells to measure maximal lysis (containing target cells with 3% Triton X-100) and spontaneous lysis (containing target cells and effector cells without antibody) were also included. Cell death was determined after 4 h of incubation at 37 °C with 5% CO_2_ by measuring LDH release using a LDH detection kit (11644793001, Roche) according to the manufacturer’s instructions. Using a kinetic protocol, the absorbance at 490 nm and 650 nm was measured once every 2 min for 8 min, and the percent specific lysis was determined. Determination of antibody-dependent cellular phagocytosis was performed using ExpiCHO transiently expressing NA from A/Perth/16/2009 (H3N2) and labelled with PKH67 (Sigma-Aldrich). Peripheral blood mononuclear cells from a healthy donor were labelled with CellTrace Violet (Invitrogen) and used as a source of phagocytic effector cells. Antibodies were serially diluted in RPMI-1640 medium. Labelled target cells (1 × 10^4^ per well) were incubated with the diluted mAbs for 10 min and then mixed with labelled PBMCs (1.8 × 10^5^ per well). Following an overnight incubation at 37 °C and 5% CO_2_, cells were stained for 20 min with APC-labelled anti-CD14 mAb (BD Pharmingen), BV605-labelled anti-CD16 mAb (BioLegend), BV711-labelled anti-CD19 mAb (BioLegend), PerCP/Cy5.5-labelled anti-CD3 mAb (BioLegend) and APC/Cy7-labelled anti-CD56 mAb (BioLegend) for the identification of CD14^+^ monocytes. After the incubation, cells were washed and fixed with 4% paraformaldehyde before acquisition on a ZE5 Cell Analyzer (Bio-Rad). Data were analysed using FlowJo software. The percentage of antibody-dependent cellular phagocytosis was calculated as the percentage of monocytes (CD14^+^ cells) positive for PKH67. The assays were performed on two different donors and the area under the curves were calculated.

### Cryo-EM sample preparation

A summary of sample preparation is provided in Supplementary Table 5. In brief, NA proteins were mixed with a 1.3–1.5-fold molar excess of FNI Fabs and incubated on ice for 1 h. Complexes were purified by size-exclusion chromatography using a Superdex 200 10/300 GL Increase (Cytiva) column equilibrated in 50 mM Tris-HCl (pH 8.0), 150 mM NaCl and 10 mM CaCl_2_. Complex containing fractions were pooled and concentrated using Amicon Ultra centrifugal filters (Millipore Sigma) before vitrification. Of 0.2–0.25 mg ml^−1^ of each NA–Fab complex with or without *n*-dodecyl-β-maltoside (DDM) at 0.70× critical micelle concentration (CMC), 3 µl were loaded onto a freshly glow discharged 1.2/1.3 UltrAuFoil grids before plunge freezing using a Vitrobot MarkIV (Thermo Fisher Scientific) with a blot force of 3 and a blot time of 7 s at 100% humidity and 4 °C.

### Cryo-EM data collection

The FNI9–NA (N2 A/Tanzania/205/2010) dataset was collected on a 300 kV Titan Krios equipped with a Gatan K3 direct detector and Gatan BioQuantum energy filter, operated with a slit width of 30 eV. Semi-automated data collection was carried out using SerialEM^57^ at a nominal magnification of ×81,000 in super-resolution mode giving a pixel size of 0.53 Å. The dose was 48 e^−^ Å^−^^2^, fractionated over 40 frames of 75 ms each.

For other datasets, data were acquired on either a 200 kV FEI Glacios transmission electron microscope (TEM) equipped with a Falcon 4 camera, or a 300 kV FEI Titan Krios TEM equipped with a Gatan K3 Summit direct detector and Gatan Quantum GIF energy filter, operated in zero-loss mode with a slit width of 20 eV. Automated data collection was carried out using Leginon^58^ at a nominal magnification of ×150,000 and a pixel size of 0.927 Å for the Glacios datasets, and ×105,000 magnification and a pixel size of 0.823 Å for the Krios datasets. The dose rate was adjusted to 1.4 counts per pixel per second, and each movie was fractionated in 24 frames for 110 ms for Glacios datasets, and 35 or 38 frames for 40 ms for the Krios datasets.

For each dataset, between 1,089 and 5,433 movies were collected with a defocus range comprised between −1.0 and −2.5 μm. A summary of data collection is provided in Supplementary Table 5.

### Cryo-EM data processing

For the FNI9–NA (N2 A/Tanzania/205/2010) dataset, Relion^59,60^ was used for cryo-electron microscopy (cryo-EM) data processing. Dose-weighted movie frame alignment was done using a Relion implementation of MotionCor2 (ref. ^61^) to account for stage drift and beam-induced motion. The contrast transfer function (CTF) was estimated for each micrograph using CTFFIND4 (ref. ^62^). A Laplacian-of-Gaussian algorithm was used for template-free automated particle picking. Extracted particles were subjected to 2D classification and only those contributing to detailed views of a NA–Fab average were used for further analysis. An initial 3D map was generated ab initio from all selected particles using Relion. An NA–Fab complex with four Fabs bound was used as input to 3D classification where 3-Fab and 4-Fab complexes were separated. These separate 3D classes were submitted to further refinement that included CTF refinement, Bayesian polishing and masking. No symmetry (C1) was applied for the further refinement of the NA–3-Fab map, but C4 symmetry was applied for the NA–4-Fab refinement.

For all other datasets, dose-weighted movie frame alignment was done using MotionCor2 (ref. ^61^) or full-frame or Patch motion correction in cryoSPARC^63^ to account for stage drift and beam-induced motion. The CTF was estimated for each micrograph using CTFfind4 (ref. ^62^), gCTF^64^ or Patch CTF in cryoSPARC. Individual particles are selected using automated picking protocols and extracted into particle stacks in either Relion^59,60^ or cryoSPARC. Extracted particles and/or 2D classes showing intact particles from Relion 3.0 were subjected to 2D and/or 3D classification in cryoSPARC. Initial maps were generated ab initio from all selected particles. The best 3D classes were submitted to homogeneous 3D refinement that included dynamic masking and either C1 (for 1-Fab or 3-Fab bound NAs) or C4 (for 4-Fab bound NAs) symmetry applied.

Reported resolutions are based on the gold-standard Fourier shell correlation of 0.143 criterion^65^. A summary of cryo-EM data statistics is provided in Supplementary Table 5.

### Cryo-EM model building and analysis

Atomic model of N2 NA from A/Tanzania/205/2010 H3N2 (Protein Data Bank (PDB) ID: 4GZX) or NA from B/Phuket/3073/2013 (PDB: 6V4O) were used as the initial model to fit into cryo-EM maps by UCSF Chimera^66^. Coot^67^ and ISOLDE^68^ were used to manually rebuild amino acid mutations and glycans on NAs and build the Fabs. Models were self-restrained by ProSMART^69^ and refined by Refmac Servalcat^70^ in the ccpem v1.6.0 software suite^71^. Model validation and map-model FSC were generated by validation: model tool in ccpem v1.6.0.

Figures were generated using Pymol^72^. Epitopes in all NA complexes were identified by determining NA residues within 5.0 Å of any atoms in FNI Fabs, 1G01, SA or oseltamivir using MOE (v2020.0901; https://www.chemcomp.com) contact analysis with default settings. MOE QuickPrep was used to prepare the NA complexes for static epitope analysis.

### Glycan profiling of NA with peptide mapping LC–MS

Peptide mapping with liquid chromatography–mass spectrometry (LC–MS) was used to profile the site-specific glycosylation sites on two NAs (A/Tanzania/205/2010 and A/Hong Kong/2671/2019). Glycopeptides containing only one specific glycan were achieved by selectively digesting with trypsin, Glu-C, Lys-C or Asp-N protease, depending on the sequence context. Of each digest product (peptide with a single glycan), 25 µg was analysed by LC–MS (Agilent AdvanceBio peptide mapping column and Thermo Q Exactive Plus Orbitrap MS). Peptide mapping data were analysed on Biopharma Finder 3.2 data analysis software. Technical replicates were performed by injecting 25 µg of digested product three times from the same sample vial into the LC–MS.

### MD methods

The coordinates of FNI17–NA (Tanzania/2010) and FNI9–NA (Tanzania/2010) were obtained from cryo-EM (see above) with glycans determined by peptide mapping LC–MS (Extended Data Fig. 7) modelled using ISOLDE as previously described^68,73^. The resulting model was then prepared using QuickPrep (MOE v2020.0901; https://www.chemcomp.com).

The FNI9–NA tetramer and FNI17–NA tetramer complex structures were each parameterized for MD using tleap in AMBER^74^ with the ff14SB protein force field^75^, the GLYCAM_06j-1 glycan force field^76^ and the TIP3P water force field^77^; the Joung and Cheatham force field^78^ was used for ions.

We generated 11.5 μs of aggregate MD for each of the two FNI–NA complexes—23 μs in total—by performing ten independent MD simulations each seeded with different initial velocities. Each of these 20 simulations was minimized and equilibrated as previously described^79^. Specifically, all heavy atoms resolved in the experimental data were restrained: during minimization, 100 ps of MD heating to 300 K; 100 ps of MD at 300 K; 250 ps of MD with a tenfold weaker restraint force constant at 300 K. With all backbone atoms resolved in the structure restrained: 10,000 steps of minimization; 100 ps of MD at 300 K; 100 ps of MD at 300 K (tenfold weaker restraint force constant); 100 ps of MD at 300 K with a further tenfold reduced force constant; 100 ps of MD at 300 K with another tenfold reduced force constant (0.1 kcal per mol Å^2^). Finally, 2.5 ns of unrestrained MD at 300 K. Production MD involved 0.8 μs of unrestrained MD simulation for each of the 20 independent simulations, followed by an additional 0.7 μs for five of the FNI9–NA and five of the FNI17–NA simulations for an aggregate of 11.5 μs of MD for FNI17–NA and 11.5 μs of MD for FNI9–NA.

Simulations were processed using cpptraj^80^ as previously described^79^. Epitope–paratope interactions were computed using contact analysis in MOE (CCG MOE 2020.09) in which all hydrogen bond, metal, ionic, arene and distance-based interactions (within 5 Å) were accounted for. This analysis was performed on MD frames sampled every 10 ns. The MOE energy scores reported are the average energy for each interaction pair in the aggregate MD. The percent occupancy reported reflects the percentage of frames in the 11.5-μs MD simulation datasets in which a particular contact was present.

### In vivo studies

Prophylactic efficacy of muFNI9 and muFM08 in BALB/C mice infected with A/Puerto Rico/8/34 was assessed at Burleson Research Technology Inc. (North Carolina) following approval of animal procedures by the Testing Facility’s Institutional Animal Care and Use Committee (IACUC). Seven-to-eight-week-old BALB/c female mice (Charles River Laboratories) received a single dose of muFNI9, muMEDI8852, muFNI9(N297Q), muMEDI8852(N297Q) or vehicle control on day −1 via intravenous injection, 24 h before infection, based on day −2 individual body weight. On day 0, animals (*n* = 6 per group) were infected intranasally with influenza A/Puerto Rico/8/34 virus (VR-95PQ, ATCC). Mice were monitored once daily from days 0–3, and twice daily from days 4–14. Body weight was measured daily from day 4 to day 14, and serum was collected at day 0 (pre-infection) for mAb quantitation.

The prophylactic activity of MEDI8852 and FNI17 mAbs administered singly or in a 1:1 combination on the response to H1N1 A/Puerto Rico/8/34 infection or human MEDI8852, FNI19 and muMEDI8852 and muFNI9 mAbs on the response to H3N2 A/Hong Kong/1/1968 was assessed in BALB/c female mice at 0.25 or 0.125 mg kg^−1^. Body weight loss and survival from days 0 through to day 14 post-infection were used as major end point. The area of the negative peaks, defined as the area between the body weight line of each animal over the 14 days and the 0% body weight loss baseline, was calculated using GraphPad prism 9 and used to compare the prophylactic activity of the anti-HA stem, anti-NA and the combination of the two mAbs.

The prophylactic efficacy of FNI9 to influenza B/Victoria/504/2000 and B/Brisbane/60/2008 was assessed in **7**–8-week-old BALB/c female mice by administering a single dose of FNI9 or vehicle control on day −1 via intravenous injection, 24 h before infection. Mice in the oseltamivir (MedChemExpress) treatment groups received 10 mg kg^−1^ of small drug via oral gavage 2 h pre-infection, 6 h post-infection and then once daily until day 3 post-infection based on day −2 individual body weight. Animals were infected intranasally with influenza B/Victoria/504/2000 or influenza B/Brisbane/60/2008 (ViraPur) virus on day 0. Serum was collected 2 h before infection on day 0 for IgG quantitation. Animals were euthanized on day 3 and lung and body weights were recorded. Infectious virus titres were measured in lung homogenate supernatants collected on day 3 (PFU per gram of lung) using a plaque assay.

The in vivo prophylactic studies involving human FNI9 IgG mAb and A/Puerto Rico/8/34 (PR8) and A/Singapore/INFHH-16-19/2016 viruses were performed in compliance with federal laws and institutional guidelines and have been approved by the Washington University in St. Louis IACUC. Seven-to-nine-week-old female BALB/c mice were randomized based on age before antibody treatment and virus infection. Anti-NA mAbs were administered as a single intravenous injection 24 h before virus infection. For oseltamivir treatment, the mice were orally dosed with 10 mg kg^−1^ of oseltamivir phosphate 2 h before infection and then once daily for 5 days. On the day of infection, mice were anaesthetized with 2% isoflurane and intranasally infected with 5 times the lethal dose 50 (5 LD_50_) of viruses diluted in 50 μl of PBS. After infection, mice were monitored daily, and their weights were recorded. To determine lung viral titres, mice were killed 4 days post-infection. Lungs were harvested and stored at −80 °C. Tissues were homogenized in 1 ml of MEM, and serial dilutions of homogenates were incubated on MDCK-LN cells for 1 h at 37 °C followed by two PBS washes. Of infection medium (MEM with 100 U ml^−1^ penicillin, 50 μg ml^−1^ streptomycin, 100 µg ml^−1^ kanamycin and 0.5 μg ml^−1^ TPCK-treated trypsin), 200 µl was added to the cells. After 3 days of incubation at 37 °C, 50 µl of supernatants were removed and added to 50 µl of 0.5% turkey red blood cells and the end point dilution of haemagglutination was scored. TCID_50_ titres were calculated using the Spearman–Karber formula.

IgG quantification was performed using an electrochemiluminescence-based immunoassay. Standard curve and quality controls were diluted in BALB/C mouse serum (Biovit) and stored at −20 °C in aliquots until needed for the quantitation assay. Reagents and samples were equilibrated to room temperature for at least 30 min. Sera, standard curve and QCs were then centrifuged at 1,000*g* for 5 min before use. For the murine mAbs quantification, multi-array 96-well standard plates (Meso Scale Discovery) were coated with NA protein (N9) or HA protein (H5) at 4 µg ml^−1^ or 2 µg ml^−1^, respectively. Plates were incubated overnight at 4 °C, then washed with PBS–0.05% Tween (PBS-T) and blocked with casein in PBS (Thermo Fisher) at room temperature for 2 h. Sera were diluted 1:20 with BALB/C mouse serum, followed by a 1:50 (for anti-NA mAb) or 1:100 (for anti-HA mAb) dilution with casein, that was performed for standard curve and QCs as well. Plates were washed with PBS-T, then 50 µl of diluted samples, standard curve and QCs were added to the appropriate wells and incubated for 1 h at room temperature. Goat anti-mouse-SULFO TAG (Meso Scale Diagnostics) was used for detection at a concentration of 1 µg ml^−1^ for FNI9 and 0.75 µg ml^−1^ for muMEDI8852 to the plates previously washed with PBS-T and incubated for 1 h. After final washes with PBS-T, 150 µl of MSD Read buffer was added per well, and plates were read on an MESO Quickplex SQ 120. Human mAbs were quantified with the same method but using an anti-LS antibody for capture and anti-CH2-SULFO TAG for detection.

### Deep mutational scanning profiling

A deep mutational scanning approach was used to determine how NA amino acid mutations affect mAb binding. A cell-surface display library based on N1 A/California/07/2009 was generated with all variants for 170 select amino acids included. The mutated residues include epitope residues within 8.0 Å distance from bound Fabs obtained from cryo-EM (FNI17–Fab and FNI19–Fab in complex with N2 A/Tanzania/205/2010; this study) and crystal structures (FNI3–Fab with N2 A/Tanzania/205/2010 (unpublished), 1E01–Fab (PDB: 6Q20), Mem5–Fab (PDB: 2AEP) and B10–Fab (PDB: 6N6B)). In addition, residues with known sequence variation in GISAID in the past 20 years for H1N1 and H5N1 were included.

Library construction, lentivirus production, next-generation sequencing and data deconvolution were performed by the Genetic Perturbation Platform (GPP) of the Broad Institute of MIT and Harvard. A detailed description of each of these methods has been previously described^81^. For library synthesis, the lentiviral vector pMT025 encoding the wild-type N1 A/California/07/2009 open reading frame with the addition of a C-terminal minimal HA tag via a 2×G_4_S linker was synthesized and used as a template to generate the variant library pool. The full-length variant pool with 5′ and 3′ flanking adapters containing NheI and MluI, respectively, was synthesized at Twist Biosciences. Following restriction digest cloning and pDNA amplification, the variant composition of the pDNA library was assessed by NGS using the Illumina Nextera XT platform. All 3,472 designed variants were detected, and the distribution of the variants was approximately log-normal with a 1.3-fold standard deviation in the read counts across the variants. Lentiviral particles were produced according to protocols available through the GPP (http://www.broadinstitute.org/rnai/public/resources/protocols). The library was transduced into FreeStyle 293-F Cells (Thermo Fisher Scientific) at a MOI of approximately 0.3 and maintained at an average representation of 2,000 cells per variant.

For the screen, mAb binding to cell-surface-displayed NA was determined by flow cytometry. mAbs were directly conjugated to Alexa Fluor 647 (Thermo Fisher Scientific) through free amine conjugation to enable fluorescence-based detection. 293-F cells containing the NA variant library were harvested 5–7 days after puromycin selection, washed in PBS containing 3% BSA and then incubated with Alexa Fluor 647-conjugated mAbs for 1 h at 4 °C in the dark. Cells were washed and fixed with 4% paraformaldehyde, then sorted in technical duplicate on two separate sorters (BD Aria Fusion or Sony MA900). Dead cells were excluded from the analysis using the LIVE/DEAD Fixable Blue Dead Cell Stain Kit (Thermo Fisher Scientific). A minimum of 60 million cells were prepared for FACS per technical replicate. 293-F cells expressing the NA variant library were sorted by FACS into negative, low and high mAb-binding bins, and the distribution of variants in each bin was determined by NGS as described below. The NA open reading frame includes a minimal C-terminal HA tag, which was also screened against using the Alexa Fluor 488 anti-HA.11 epitope tag antibody (BioLegend) to determine how mutations at each of the 170 selected residues affect general protein expression. For each screen, a minimum of 5 million unsorted library cells were collected to serve as unsorted controls.

Genomic DNA was isolated from sorted samples and unsorted controls with the QIAamp DNA FFPE Tissue Kit (with the following modification: 0.3 M NaCl was added to the lysis buffer and incubation at 56 °C extended to 4 h) and the QIAamp DNA Blood Kit (Qiagen), respectively. Genomic DNA was prepared for NGS as previously described^81^. In brief, the open reading frame was amplified from genomic DNA by PCR with the following primers (forward: 5′-ATTCTCCTTGGAATTTGCCCTT-3′ and reverse: 5′-CATAGCGTAAAAGGAGCAACA-3′) and processed for NGS using the Illumina Nextera XT kit. Samples were sequenced using the Nextseq2000 sequencing platform to obtain paired-end reads with 2 × 150 cycles. Sequence data processing and variant calling using the mutant detection software ASMv1.0 was performed as previously described^81^. Normalized reads for each sample are log_2_ transformed. log_2_ fold-change values comparing the negative sorted bin to the unsorted pool and the low-binding and high-binding FACS-sorted bins for each library variant were computed and then averaged both for the anti-HA tag and the anti-NA antibody. Potential escapes were defined as those having an HA tag signal close to the silent mutations (maximum of two standard deviations away from the mean of the silent mutations) and having an anti-NA signal close to the stop codon mutations (maximum of three standard deviations away from the mean of the stop codon mutations).

### Sequence conservation analysis

H3N2 (IAV), H1N1 (IAV), H5N1 (IAV), H7N9 (IAV), H5N8 (IAV), H5N6 (IAV), Victoria (IBV) and Yamagata (IBV) NA protein sequences were retrieved from GISAID (www.gisaid.org). Protein sequences were aligned to a reference NA sequence using MAFFT^82^. For Fig. 2, the sequences used were retrieved from the GISAID repository from January 2000 to October 2022, and the respective reference used for alignment was A/California/07/2009 (NC_026434.1) for H1N1 sequences, A/NewYork/392/2004 (YP_308842.1) for H3N2 sequences and B/Yamagata/16/1988 (AAN39803.1) for Victoria and Yamagata IBV sequences. For Extended Data Fig. 5, the sequences used were retrieved in July 2022 and the respective reference used for alignment was A/NewYork/392/2004 (YP_308842.1) for H3N2, H1N1, H5N1, H7N9 H5N8 and H5N6 sequences, and B/Yamagata/16/1988 (AAN39803.1) for Victoria and Yamagata IBV sequences. The multiple sequence alignments were analysed with R (https://www.R-project.org/) v.4.0.4. The logo plots were generated with the R package ‘ggseqlogo’ v.0.1 (ref. ^83^). The conservation per residue was computed with the R package ‘Biostrings’ v.2.58.0 (https://bioconductor.org/packages/Biostrings).

### Statistical analysis

All statistical tests were performed as described in the indicated figure legends using Prism v9.0. The number of independent experiments performed is indicated in the relevant figure legends.

### Material availability

Materials generated in this study can be available on request from the corresponding authors and may require a material transfer agreement.

### Ethical statement

All experiments involving animals reported in Fig. 5a–d,g,h and Extended Data Fig. 9 were approved by the Burleson Research Technologies, Inc. (BRT) Animal Care and Use Committee (study number: BRT20220306, BRT 2022072 and BRTQ20220307) and by the Office of Laboratory Animal Welfare (PHS assurance number D16-00898). The BRT has been accredited by the Association for Assessment and Accreditation of Laboratory Animal Care (AAALAC) International. The IACUC chair who approved the protocols was also the Lead Veterinarian for these studies, and together with the Study Director was kept apprised of body weight loss and clinical observations for these studies daily. In addition, both the Lead Veterinarian and the Study Director personally checked on the status of study mice throughout each of the studies.

On the basis of extended experience with the influenza model at the BRT, specific weight loss limits were not set for the study reported in Fig. 5a–d and Extended Data Fig. 9 (BRT20220306 and BRTQ20220711), as mice with 30% (or greater) body weight loss had normal cage activity levels indicated by the ability to climb to access the food hoppers and build nests and to interact with cage mates as expected. In addition, mice with weight loss that exceeded 30% were shown to be able to recover from infection in this model. In such scenarios, the BRT would not euthanize the mouse but instead would wait until the next observation period to reassess behaviour along with any further body weight loss. At each observation period, both the Lead Veterinarian and the Study Director specifically checked for animals that met euthanasia requirements as listed in both the study protocol and the IACUC protocol.

Given the nature of the study and the weight loss involved, measures were undertaken to minimize pain, distress and discomfort as much as possible, including providing DietGel to aid in providing hydration and nutrition from the time of infection through to day 14, increased frequency of animal observations (twice daily, at least 5 h between observations on days 4–14 and on other days if adverse clinical signs were observed), daily body weight and clinical observation updates provided to the Study Director and veterinarian, and humane euthanasia if animals were deemed unlikely to recover from infection.

In addition to protocol-driven euthanasia requirements, although body weight loss was not used as an end point, it was used to further evaluate the mice and help to inform euthanasia decisions. If, for example, body weight loss was high and was not stabilizing and/or if body weight loss was high and other clinical signs (especially a strong decrease in movement or activity) were observed, animals were immediately humanely euthanized as either scenario was generally a good indication that the mouse was unlikely to recover from infection.

Procedures, facilities and housing were in compliance with the NRC Guide for the Care and Use of Laboratory Animals. Mice were euthanized at 3 days post-infection (Fig. 5g,h; BRT 2022072) or throughout a 14-day observation period (Fig. 5a–d and Extended Data Fig. 9; BRT20220306 and BRTQ20220711) according to the AVMA Guidelines for the Euthanasia of Animals: 2020 edition and in compliance with all applicable state and federal laws.

All experiments involving animals reported in Fig. 5e,f were approved by Washington University in St. Louis IACUC (Animal Welfare Assurance D16-00245). All facilities involving animal research were accredited by the Association for Assessment and Accreditation of Laboratory Animal Care International. Procedures, facilities and housing were in compliance with the NRC Guide for the Care and Use of Laboratory Animals. Mice were euthanized at 4 days post-infection according to the AVMA Guidelines for the Euthanasia of Animals: 2020 edition and in compliance with all applicable state and federal laws.

A power analysis (80%) with type 1 error set at 5% based on an estimated effect size and historical data were used to determine animal numbers for experiments and to keep the number of animals used to a minimum.

### Reporting summary

Further information on research design is available in the Nature Portfolio Reporting Summary linked to this article.

## Online content

Any methods, additional references, Nature Portfolio reporting summaries, source data, extended data, supplementary information, acknowledgements, peer review information; details of author contributions and competing interests; and statements of data and code availability are available at 10.1038/s41586-023-06136-y.

## Supplementary information

Reporting Summary

Supplementary Table 1 **HCDR3 and LCDR3 sequences and percentage of homology to UCA of clonally related FNI mAbs**. This table summarizes the percentage of homology of the FNI mAbs VH and VL regions to unmutated common ancestor (UCA) and reports for each mAb the amino acid sequences of the heavy chain and light chain complementarity-determining regions (HCDR3 and LCDR3).

Supplementary Table 2 **List of NA constructs transiently expressed in mammalian cells assessed for FNI9, FNI17, and FNI19 mAbs binding by FACS**. This table reports the subtypes, strains, GISAID and/or GenBank accession numbers and relevant references for the NA antigens tested by FACS with FNI9, FNI17, and FNI19 mAbs as shown in Fig.1b.

Supplementary Table 3 **List of the IAV/IBV strains and corresponding neutralization IC**_**50**_ **values for the anti-NA mAbs related to Fig. 2a**. This table shows the full designation of the strains listed as abbreviations in Fig.2a and the corresponding neutralization IC50 values for FNI9, FNI17, FNI19, and 1G01 mAbs.

Supplementary Table 4 **List of NA antigens used in the study and corresponding binding affinity values (KD) for FNI9, FNI17, FNI19 and 1G01 Fabs as measured by SPR**. This table summarises the binding affinity measure by surface plasmon resonance (SPR) for FNI9, FNI17, FNI19, and 1G01 Fabs versus the N1 or N2 antigens described in the article.

Supplementary Table 5 **Cryo-EM data collection, refinement and validation statistics** This table summarises the data collection, refinement and validation statistics for the Cryo-EM structures presented in the article.

## Data Availability

The cryo-EM maps and atomic coordinates have been deposited to the Electron Microscopy Data Bank (EMDB) and the PDB with accession numbers: 8G3M and EMD-29704 for 3-FNI9-Fab-bound or 8G3N and EMD-29705 for 4-FNI9-Fab-bound NA from the A/Tanzania/205/2010 strain; 8G3O and EMD-29706 for 3-FNI9-Fab-bound or 8G3P and EMD-29707 for 4-FNI9-Fab-bound NA from the A/Hong_Kong/2671/2019 strain; 8G3Q and EMD-29708 for 3-FNI17-Fab-bound NA from the A/Tanzania/205/2010 strain; 8G3R and EMD-29709 for 1-FNI17-Fab-bound NA from the A/Tanzania/205/2010 strain with S245N and S247T mutations; 8G30 and EMD-29686 for 4-FNI19-Fab-bound NA from the A/Tanzania/205/2010 H3N2 strain; 8G40 and EMD-29712 for 3-FNI19-Fab-bound or 8G3V and EMD-29710 for 4-FNI19-Fab-bound NA from the A/Hong_Kong/2671/2019 strain; and 8G3Z and EMD-29711 for 4-FNI17-Fab-bound NA from the B/Massachusetts/02/2012 (Yamagata) strain. A summary of samples with corresponding EMDB and PDB accession codes can be found in Supplementary Table 5. All datasets generated and information presented in the study are available from the corresponding authors on reasonable request. Materials generated in this study can be available on request and may require a material transfer agreement. Source data are provided with this paper.

## Source data

Source Data Fig. 2
Source Data Fig. 3
Source Data Fig. 4
Source Data Fig. 5
Source Data Extended Data Fig. 2
Source Data Extended Data Fig. 3
Source Data Extended Data Fig. 4
Source Data Extended Data Fig. 5
Source Data Extended Data Fig. 6
Source Data Extended Data Fig. 7
Source Data Extended Data Fig. 8
Source Data Extended Data Fig. 9
Source Data Extended Data Fig. 10
