# Author Correction: A pan-influenza antibody inhibiting neuraminidase via receptor mimicry

**DOI:** 10.1038/s41586-023-06385-x

**Published:** 2023-07-05

**Authors:** Corey Momont, Ha V. Dang, Fabrizia Zatta, Kevin Hauser, Caihong Wang, Julia di Iulio, Andrea Minola, Nadine Czudnochowski, Anna De Marco, Kaitlin Branch, David Donermeyer, Siddhant Vyas, Alex Chen, Elena Ferri, Barbara Guarino, Abigail E. Powell, Roberto Spreafico, Samantha S. Yim, Dale R. Balce, Istvan Bartha, Marcel Meury, Tristan I. Croll, David M. Belnap, Michael A. Schmid, William Timothy Schaiff, Jessica L. Miller, Elisabetta Cameroni, Amalio Telenti, Herbert W. Virgin, Laura E. Rosen, Lisa A. Purcell, Antonio Lanzavecchia, Gyorgy Snell, Davide Corti, Matteo Samuele Pizzuto

**Affiliations:** 1 Vir Biotechnology, San Francisco, CA USA; 2 Humabs Biomed SA, a subsidiary of Vir Biotechnology, Bellinzona, Switzerland; 3 Vir Biotechnology, St. Louis, MO USA; 4 Cambridge Institute for Medical Research, Department of Haematology, University of Cambridge, Cambridge, UK; 5 School of Biological Sciences, Department of Biochemistry, University of Utah, Salt Lake City, UT USA; 6 Department of Pathology and Immunology, Washington University School of Medicine, St. Louis, MO USA

**Keywords:** Influenza virus, Influenza virus

Correction to: *Nature* 10.1038/s41586-023-06136-y Published online 31 May 2023

In the version of this article initially published, in the Data availability section, an incorrect Electron Microscopy Data Bank accession number (EMD-29703) was listed alongside the PDB entry 8G3M, which has now been corrected to read “EMD-29704” in the HTML and PDF versions of the article.

