# Supplementary material for: A pan-influenza antibody inhibiting neuraminidase via receptor mimicry
Source: Nature. 2023 May 31;618(7965):590–7. doi: 10.1038/s41586-023-06136-y (PMC10266979; doi:10.1038/s41586-023-06136-y)
Supplement: Supplementary file 1 — Reporting Summary [file 41586_2023_6136_MOESM1_ESM.pdf]

nature portfolio

Corresponding author(s): Snell, Corti, Pizzuto

Last updated by author(s): Apr 11, 2023

Reporting Summary

Nature Portfolio wishes to improve the reproducibility of the work that we publish. This form provides structure for consistency and transparency in reporting. For further information on Nature Portfolio policies, see our Editorial Policies and the Editorial Policy Checklist.

Statistics

For all statistical analyses, confirm that the following items are present in the figure legend, table legend, main text, or Methods section.

n/a	Confirmed	
☐	☒	The exact sample size (*n*) for each experimental group/condition, given as a discrete number and unit of measurement
☐	☒	A statement on whether measurements were taken from distinct samples or whether the same sample was measured repeatedly
☐	☒	The statistical test(s) used AND whether they are one- or two-sided<br>*Only common tests should be described solely by name; describe more complex techniques in the Methods section.*
☒	☐	A description of all covariates tested
☒	☐	A description of any assumptions or corrections, such as tests of normality and adjustment for multiple comparisons
☐	☒	A full description of the statistical parameters including central tendency (e.g. means) or other basic estimates (e.g. regression coefficient) AND variation (e.g. standard deviation) or associated estimates of uncertainty (e.g. confidence intervals)
☒	☐	For null hypothesis testing, the test statistic (e.g. *F*, *t*, *r*) with confidence intervals, effect sizes, degrees of freedom and *P* value noted<br>*Give P values as exact values whenever suitable.*
☒	☐	For Bayesian analysis, information on the choice of priors and Markov chain Monte Carlo settings
☒	☐	For hierarchical and complex designs, identification of the appropriate level for tests and full reporting of outcomes
☒	☐	Estimates of effect sizes (e.g. Cohen's *d*, Pearson's *r*), indicating how they were calculated

Our web collection on statistics for biologists contains articles on many of the points above.

Software and code

Policy information about availability of computer code

Data collection

As described in Methods:
* protein purification monitored by UNICORN software version 5.11
* SPR binding assays collected and analyzed using Biacore Evaluation and Biacore Insight software
* FACS data collected using ZE5 Flow Cytometer (Biorad)
* MUNANA and ELLA data were obtained using Cytation5 (Biotek) and Powerwave 340/96 spectrophotometer (Biotek).
* NP-staining neutralization was imaged by a Cytation5 plate reader from Agilent.
* Cryo-EM: data was acquired by SerialEM (v.3.8) or Leginon (v.3.6).
* Molecular dynamics (MD) input structures prepared using MOE (v2020.0901, https://www.chemcomp.com), parameters generated, simulations computed and processing executed using AMBER20.
* Epitope conservation analysis: H3N2 (IAV), H1N1 (IAV), H5N1 (IAV), H7N9 (IAV), H5N8 (IAV), H5N6 (IAV), Victoria (IBV) and Yamagata (IBV) NA protein sequences were retrieved from GISAID (www.gisaid.org).
DMS NGS samples were sequenced using the Nextseq2000 sequencing platform.

Data analysis

Binding, MUNANA, ELLA, Neutralization assays analyzed using PRISM (Version 9.1.0) as described in Methods.
FACS data analysed with FlowJo software.
SPR binding assays: Biacore Evaluation (version 3.2.1) and Biacore Insight software (version 4.0.8.20368).
Crystallography data analysis performed with XDS (VERSION Jan 31, 2020 BUILT=20200417), Coot (v. 0.9.5), ChimeraX (v. 1.1)/ISOLDE (v. 1.1), Refmac5 (v. 5.8.0267), and MOE (v. 2019.0102).
Cryo-EM: RELION (v.3.1, v.3.1.1 and v4.0-beta2), MotionCor2, CTFFIND4, cryoSPARC (v2, v3.3), gCTF.
Molecular Dynamics (MD) analysis used MOE (v2020.0901, https://www.chemcomp.com).
Epitope conservation analysis: protein sequences were aligned to a reference NA sequence using MAFFT [PMID: 23329690]. For Figure 3, the

sequences used were retrieved in October 2022, and the respective reference used for alignment was A/California/07/2009 (NC_026434.1) for H1N1 sequences, A/NewYork/392/2004 (YP_308842.1) for H3N2 sequences and B/Yamagata/16/1988 (AAN39803.1) for Victoria and Yamagata IBV sequences. For Extended Data Fig. 5, the sequences used were retrieved in July 2022 and the respective reference used for alignment was A/NewYork/392/2004 (YP_308842.1) for H3N2, H1N1, H5N1, H7N9 H5N8, H5N6 sequences, and B/Yamagata/16/1988 (AAN39803.1) for Victoria and Yamagata IBV sequences. The multiple sequence alignments were analysed with R (https://www.R-project.org/) v.4.0.4. The logo plots were generated with R package "ggseqlogo" v.0.1 [PMID: 29036507]. The conservation per residue was computed with the R package "Biostrings" v.2.58.0 (https://bioconductor.org/packages/Biostrings).
DMS library design and NGS sequence analysis code is made available by the GPP on GitHub, specifically: library designer (VariantLibrary_Designer_v1.9.R, https://github.com/broadinstitute/SatMut_VariantLibrary_Designer/releases/tag/v1.9), variant-calling software (ASMv1.0 as a part of the Genome Analysis Toolkit v4.2.0.0, https://github.com/broadinstitute/gatk/releases), and data analysis tools (ASM_parser.R, https://github.com/broadinstitute/SatMut_ASM_Parser/releases/tag/v1.9).

For manuscripts utilizing custom algorithms or software that are central to the research but not yet described in published literature, software must be made available to editors and reviewers. We strongly encourage code deposition in a community repository (e.g. GitHub). See the Nature Portfolio guidelines for submitting code & software for further information.

Data

Policy information about availability of data

All manuscripts must include a data availability statement. This statement should provide the following information, where applicable:

- Accession codes, unique identifiers, or web links for publicly available datasets
- A description of any restrictions on data availability
- For clinical datasets or third party data, please ensure that the statement adheres to our policy

All datasets generated and information presented in the study will be available from the corresponding author on reasonable request.
The cryoEM maps and atomic coordinates have been deposited to Electron Microscopy Data Bank (EMDB) and Protein Data Bank (PDB) with accession numbers: 8G3M and EMD-29704 for 3-FNI9-Fab-bound or 8G3N and EMD-29705 for 4-FNI9-Fab-bound NA from A/Tanzania/205/2010 strain; 8G3O and EMD-29706 for 3-FNI9-Fab-bound or 8G3P and EMD-29707 for 4-FNI9-Fab-bound NA from A/Hong_Kong/2671/2019 strain; 8G3Q and EMD-29708 for 3-FNI17-Fab-bound NA from A/Tanzania/205/2010 strain; 8G3R and EMD-29709 for 1-FNI17-Fab-bound NA from A/Tanzania/205/2010 strain with S245N-S247T mutations; 8G30 and EMD-29686 for 4-FNI19-Fab-bound NA from A/Tanzania/205/2010 H3N2 strain; 8G40 and EMD-29712 for 3-FNI19-Fab-bound or 8G3V and EMD-29710 for 4-FNI19-Fab-bound NA from A/Hong_Kong/2671/2019 strain; and 8G3Z and EMD-29711 for 4-FNI17-Fab-bound NA from B/Massachusetts/02/2012 (Yamagata) strain. A summary of samples with corresponding EMDB and PDB accession codes can be found in Supplementary Table 5.
Molecular Dynamics inputs and scripts are available from the corresponding author on reasonable request.
Epitope conservation analysis are available from the corresponding author on reasonable request.
NGS data are available upon request.

Human research participants

Policy information about studies involving human research participants and Sex and Gender in Research.

Reporting on sex and gender	*Use the terms sex (biological attribute) and gender (shaped by social and cultural circumstances) carefully in order to avoid confusing both terms. Indicate if findings apply to only one sex or gender; describe whether sex and gender were considered in study design whether sex and/or gender was determined based on self-reporting or assigned and methods used. Provide in the source data disaggregated sex and gender data where this information has been collected, and consent has been obtained for sharing of individual-level data; provide overall numbers in this Reporting Summary. Please state if this information has not been collected. Report sex- and gender-based analyses where performed, justify reasons for lack of sex- and gender-based analysis.*
Population characteristics	*Describe the covariate-relevant population characteristics of the human research participants (e.g. age, genotypic information, past and current diagnosis and treatment categories). If you filled out the behavioural & social sciences study design questions and have nothing to add here, write "See above."*
Recruitment	*Describe how participants were recruited. Outline any potential self-selection bias or other biases that may be present and how these are likely to impact results.*
Ethics oversight	*Identify the organization(s) that approved the study protocol.*

Note that full information on the approval of the study protocol must also be provided in the manuscript.

Field-specific reporting

Please select the one below that is the best fit for your research. If you are not sure, read the appropriate sections before making your selection.

☒ Life sciences ☐ Behavioural & social sciences ☐ Ecological, evolutionary & environmental sciences

For a reference copy of the document with all sections, see nature.com/documents/nr-reporting-summary-flat.pdf

Life sciences study design

All studies must disclose on these points even when the disclosure is negative.

Sample size	N/A
Data exclusions	N/A
Replication	Experimental assays were performed at least in two independent replicates. Each replicate was performed with 2, 3, or more technical repeats according to or exceeding standards in the field. We conducted all neutralization and antibody functional assays in biological duplicate, triplicate, or more, as indicated in relevant figure legends. In all cases, representative figure displays were appropriately indicated.
Randomization	Randomization was not a relevant feature as we were applying a uniform set of techniques across a panel of monoclonal antibodies.
Blinding	Blinding was not a relevant feature as we were applying a uniform set of techniques across a panel of monoclonal antibodies and tests were repeated two or more times by different individuals.

Reporting for specific materials, systems and methods

We require information from authors about some types of materials, experimental systems and methods used in many studies. Here, indicate whether each material, system or method listed is relevant to your study. If you are not sure if a list item applies to your research, read the appropriate section before selecting a response.

Materials & experimental systems

n/a	Involved in the study	
☐	☒	Antibodies
☐	☒	Eukaryotic cell lines
☒	☐	Palaeontology and archaeology
☐	☒	Animals and other organisms
☒	☐	Clinical data
☒	☐	Dual use research of concern

Methods

n/a	Involved in the study	
☒	☐	ChIP-seq
☐	☒	Flow cytometry
☒	☐	MRI-based neuroimaging

Antibodies

Antibodies used	All the anti-NA and anti-HA mAbs described in the study were produced as recombinant IgG1 in mammalian cells as described in material and methods. All the commercial antibodies used in the study have been indicated in the M&M section with supplier name, catalog number. The monoclonal antibodies used as controls/comparator or in combination studies were previously described by publications indicated in the manuscript.
Validation	Target validation of this antibody was performed by multiple binding and NAI assays. In addition cryoEM structures were determined. Reactivity of commercial antibodies was based on the information on manufacturer's homepages.

Eukaryotic cell lines

Policy information about cell lines and Sex and Gender in Research

Cell line source(s)	Cell lines were obtained from ATCC (HEK293T/17, MDCK, MDCK-LN, A549), or Thermo Fisher Scientific (ExpiCHO-S and Expi293F cells).
Authentication	None of the cell lines used were authenticated
Mycoplasma contamination	Cell lines were tested for mycoplasma contamination
Commonly misidentified lines (See ICLAC register)	No commonly misidentified cell lines were used in the study

Animals and other research organisms

Policy information about studies involving animals; ARRIVE guidelines recommended for reporting animal research, and Sex and Gender in Research

Laboratory animals	7-9 weeks old BALB/c female mice were used in the in vivo studies

Wild animals	The study did not involved wild animals
Reporting on sex	Sex of the animals is not considered relevant for the studies
Field-collected samples	The study did not involved samples collected from the field
Ethics oversight	All experiments involving animals reported in Fig. 5a-d, 5g, h, and Extended Data Figure 9 were approved by the Burleson Research Technologies, Inc. (BRT) Animal Care and Use Committee (Study number: BRT20220306, BRT 2022072, and BRTQ20220307) and by the Office of Laboratory Animal Welfare (OLAW) (PHS Assurance Number D16-00898). All experiments involving animals reported in Fig. 5e, f were approved by Washington University in St. Louis Institutional Animal Care and Use Committee (Animal Welfare Assurance D16-00245). All facilities involving animal research were accredited by the Association for Assessment and Accreditation of Laboratory Animal Care International.

Note that full information on the approval of the study protocol must also be provided in the manuscript.

Flow Cytometry

Plots

Confirm that:

☒ The axis labels state the marker and fluorochrome used (e.g. CD4-FITC).

☒ The axis scales are clearly visible. Include numbers along axes only for bottom left plot of group (a 'group' is an analysis of identical markers).

☐ All plots are contour plots with outliers or pseudocolor plots.

☒ A numerical value for number of cells or percentage (with statistics) is provided.

Methodology

Sample preparation	Expi-CHO or Expi293 cells transiently expressing different NAs were harvested 48 h post-transfection and washed twice in FACS buffer (PBS, 2% FBS, 2 mM EDTA). Cells were counted and seeded in 96-w U-bottom plates (75000 cells/well). Sera or mAbs were serially diluted and incubated with the cells. Alexa Fluor 647-labelled secondary antibody was used for the detection.
Instrument	Biorad ZE5 Flow Cytometer
Software	Flowjo V10.7
Cell population abundance	Expi-CHO or Expi293 cell line transiently transfected to express NA.
Gating strategy	FSC/SSC gate was set to exclude debris/dead cells. FSC-H/FSC-W gate was set to analyze single cells only (excluding doublets). The cells transfected with empty plasmid (no NA expression) were used to define the negative population (AF647 negative cells).

☒ Tick this box to confirm that a figure exemplifying the gating strategy is provided in the Supplementary Information.
