# Supplementary material for: A pan-influenza antibody inhibiting neuraminidase via receptor mimicry
Source: Nature. 2023 May 31;618(7965):590–7. doi: 10.1038/s41586-023-06136-y (PMC10266979; doi:10.1038/s41586-023-06136-y)
Supplement: Supplementary file 2 — HCDR3 and LCDR3 sequences and percentage of homology to UCA of clonally related FNI mAbs. This table summarizes the percentage of homology of the FNI mAbs VH and VL regions to unmutated common ancestor (UCA) and reports for each mAb the amino acid sequences of the heavy chain and light chain complementarity-determining regions (HCDR3 and LCDR3). [file 41586_2023_6136_MOESM2_ESM.docx]

**Supplementary Table 1|** **HCDR3 and LCDR3 sequences and percentage of homology to UCA of clonally related FNI mAbs.**

|  | **VH** | **HCDR3** | **VL** | **LCDR3** |
| --- | --- | --- | --- | --- |
| **FNI1** | IGHV1-69 (89.58%) | ARAVSDYFNRDLGWDDYYFPL | IGKV3-15 (95.7%) | QHYNTWPPWT |
| **FNI2** | IGHV1-69 (90.97%) | ARAVSDYFNRDLGWEDYYFPI | IGKV3-15 (97.49%) | QHYNNWPPWT |
| **FNI3** | IGHV1-69 (91.32%) | ARAGSDYFNRDLGWENYYFDS | IGKV3-15 (94.98%) | QHYNNWPPWT |
| **FNI4** | IGHV1-69 (89.24%) | ARAHSDYFNRDLGWEDYYFDY | IGKV3-15 (96.77%) | QQYNNWPPWT |
| **FNI5** | IGHV1-69 (90.97%) | ARARSDYFNRDLGWDDYYFDY | IGKV3-15 (97.49%) | QQYNNWPPWT |
| **FNI6** | IGHV1-69 (92.71%) | ARAGSDYFNRDLGWENYYFEY | IGKV3-15 (93.55%) | QHYNNWPPWT |
| **FNI7** | IGHV1-69 (86.46%) | ARARSDYFNRDLGWENYYFES | IGKV3-15 (93.55%) | QHYSYWPPWT |
| **FNI9** | IGHV1-69 (89.58%) | ARAGSDYFNRDLGWENYYFAS | IGKV3-15 (94.27%) | QQYNNWPPWT |
| **FNI10** | IGHV1-69 (86.81%) | ARAVSDYFNRDLGWENYYFES | IGKV3D-15 (93.91%) | QHYNNWPPWT |
| **FNI12** | IGHV1-69 (87.5%) | ATTRSDYFNRDLGWEDYFFDH | IGKV3-15 (98.21%) | QHYNNWPPWT |
| **FNI13** | IGHV1-69 (92.01%) | ARANSDYFNRDLGWENYYFED | IGKV3-15 (94.27%) | QHYNIWPPWT |
| **FNI14** | IGHV1-69 (87.85%) | ARAVSDYFNRDLGWDDYYFPL | IGKV3-15 (95.7%) | QHYNNWPPWT |
| **FNI17** | IGHV1-69 (92.71%) | ARARSDYFNRDLGWEDYYFEN | IGKV3-15 (98.21%) | QHYNNWPPWT |
| **FNI19** | IGHV1-69 (91.32%) | ATAVSDYFNRDLGWEDYYFPF | IGKV3-15 (94.27%) | QHYNIWPPWT |
