# Supplementary material for: A pan-influenza antibody inhibiting neuraminidase via receptor mimicry
Source: Nature. 2023 May 31;618(7965):590–7. doi: 10.1038/s41586-023-06136-y (PMC10266979; doi:10.1038/s41586-023-06136-y)
Supplement: Supplementary file 3 — List of NA constructs transiently expressed in mammalian cells assessed for FNI9, FNI17, and FNI19 mAbs binding by FACS. This table reports the subtypes, strains, GISAID and/or GenBank accession numbers and relevant references for the NA antigens tested by FACS with FNI9, FNI17, and FNI19 mAbs as shown in Fig.1b. [file 41586_2023_6136_MOESM3_ESM.docx]

**Supplementary Table 2|** **List of NA constructs transiently expressed in mammalian cells assessed for FNI9, FNI17, and FNI19 mAbs binding by FACS.**

| Type |  | Subtype/ Lineage | Strain | Accession number | Reference |
| --- | --- | --- | --- | --- | --- |
| Influenza A | Group 1 | N1 | H1N1 A/California/07/2009 | EPI_ISL_29577 | ^84^ |
|  |  | N1 | H1N1 A/Stockholm/18/2007 | EPI_ISL_157400 | Seasonal strain before 2009 pandemic |
|  |  | N4 | H6N4 A/mallard duck/Netherlands/30/2011 | EPI_ISL_243398 | ^85^ |
|  |  | N5 | H6N5 A/aquatic bird/Korea/CN5/2009 | AFQ60391 | ^86^ |
|  | Group 2 | N2 | H2N2 A/Leningrad/134/17/57 | EPI_ISL_169836 | Pandemic strain 1957 |
|  |  | N2 | H3N2 A/Hong Kong/68 | ABQ97206 | ^87^ |
|  |  | N3 | H7N3 A/Canada/rv504/2004 | EPI_ISL_5873 | Similar to H7N3 HPAI WHO recommendation as vaccine candidate |
|  |  | N6 | H4N6 A/swine/Ontario/01911-1/99 | AAG17431 | ^88,89^ |
|  |  | N7 | H7N7 A/Netherlands/078/03 | EPI_ISL_90875 | ^90^ |
|  |  | N9 | H7N9 A/Anhui/1/2013 | EPI_ISL_138739 | WHO recommendation on influenza A (H7N9) vaccine virus |
| Influenza B | | Ancestral | B/Lee/10/1940 | NP_056663 | ^91^ |
|  |  | Victoria | B/Brisbane/60/2008 | ADN32819 | ^92^ |
|  |  | Victoria | B/Malaysia/2506/2004 | ACR15736 | ^92^ |
|  |  | Yamagata | B/Malaysia/3120318925/2013 | ANK57741 | ^93^ |
|  |  | Yamagata | B/Yamanashi/166/1998 | ABN50507 | ^92^ |

**Reference Related to Supplementary Table 2**

84 Garten, R. J. *et al.* Antigenic and genetic characteristics of swine-origin 2009 A(H1N1) influenza viruses circulating in humans. *Science* **325**, 197-201 (2009). https://doi.org:10.1126/science.1176225

85 Campbell, L. K. & Magor, K. E. Pattern Recognition Receptor Signaling and Innate Responses to Influenza A Viruses in the Mallard Duck, Compared to Humans and Chickens. *Front Cell Infect Microbiol* **10**, 209 (2020). https://doi.org:10.3389/fcimb.2020.00209

86 Nam, J. H. *et al.* Emergence of mammalian species-infectious and -pathogenic avian influenza H6N5 virus with no evidence of adaptation. *J Virol* **85**, 13271-13277 (2011). https://doi.org:10.1128/JVI.05038-11

87 Kobasa, D. *et al.* Amino acid residues contributing to the substrate specificity of the influenza A virus neuraminidase. *J Virol* **73**, 6743-6751 (1999). https://doi.org:10.1128/JVI.73.8.6743-6751.1999

88 Song, H. *et al.* Avian-to-Human Receptor-Binding Adaptation by Influenza A Virus Hemagglutinin H4. *Cell Rep* **20**, 1201-1214 (2017). https://doi.org:10.1016/j.celrep.2017.07.028

89 Karasin, A. I. *et al.* H4N6 influenza virus isolated from pigs in Ontario. *Can Vet J* **41**, 938-939 (2000).

90 Sheldon, T. Vet dies from pneumonia in avian flu case. *BMJ* **326**, 952 (2003). https://doi.org:10.1136/bmj.326.7396.952/h

91 Katz, G. *et al.* Morphology of influenza B/Lee/40 determined by cryo-electron microscopy. *PLoS One* **9**, e88288 (2014). https://doi.org:10.1371/journal.pone.0088288

92 Langat, P. *et al.* Genome-wide evolutionary dynamics of influenza B viruses on a global scale. *PLoS Pathog* **13**, e1006749 (2017). https://doi.org:10.1371/journal.ppat.1006749

93 Oong, X. Y. *et al.* Whole-Genome Phylogenetic Analysis of Influenza B/Phuket/3073/2013-Like Viruses and Unique Reassortants Detected in Malaysia between 2012 and 2014. *PLoS One* **12**, e0170610 (2017). https://doi.org:10.1371/journal.pone.0170610
