# Supplementary material for: A pan-influenza antibody inhibiting neuraminidase via receptor mimicry
Source: Nature. 2023 May 31;618(7965):590–7. doi: 10.1038/s41586-023-06136-y (PMC10266979; doi:10.1038/s41586-023-06136-y)
Supplement: Supplementary file 4 — List of the IAV/IBV strains and corresponding neutralization IC50 values for the anti-NA mAbs related to Fig. 2a. This table shows the full designation of the strains listed as abbreviations in Fig.2a and the corresponding neutralization IC50 values for FNI9, FNI17, FNI19, and 1G01 mAbs. [file 41586_2023_6136_MOESM4_ESM.docx]

**Supplementary Table 3. List of the IAV/IBV strains and corresponding neutralization IC_50_ values for the anti-NA mAbs related to Fig. 2a.**

|  |  |  | **In vitro neutralization IC_50_ (µg/mL)** | | | |
| --- | --- | --- | --- | --- | --- | --- |
|  |  |  | **FNI9** | **FNI17** | **FNI19** | **1G01** |
| **Strain** | **Abbreviation** | **Group** | **IC_50_** | **IC_50_** | **IC_50_** | **IC_50_** |
| A/Indiana/02/2020 | A/IN/2020 | H1N1 | 0.161 | 0.036 | 0.150 | 34.421 |
| A/Alabama/03/2020, NA: H275 | A/AL/2020 H275Y | H1N1 | 0.283 | 0.060 | 0.057 | 20.052 |
| A/Illinois/45/2019 | A/IL/2019 | H1N1 | 0.243 | 0.035 | 0.039 | 0.673 |
| A/Hawaii/66/2019 | A/HW/2019 | H1N1 | 0.039 | 0.018 | 0.021 | 0.306 |
| A/Victoria/2570/2019 | A/VC/2019 | H1N1 | 0.168 | 0.036 | 0.071 | 15.054 |
| A/Idaho/07/2018 | A/ID/2018 | H1N1 | 0.079 | 0.022 | 0.043 | 0.293 |
| A/Michigan/45/2015 | A/MC/2015 | H1N1 | 0.151 | 0.073 | 0.062 | 0.570 |
| A/California/2009 | A/CA/2009 | H1N1 | 0.147 | 0.056 | 0.094 | 0.147 |
| A/Puerto Rico/1934 | A/PR8/1934 | H1N1 | 0.028 | 0.011 | 0.012 | 4.809 |
| A/HongKong/4801/2014 | A/HK/2014 | H3N2 | 0.022 | 0.208 | 0.122 | 0.058 |
| A/Washington/33/2014, NA:E119V | A/WA/2014 E119V | H3N2 | 0.079 | 0.219 | 0.091 | 0.047 |
| A/Switzerland/9715293/2013 | A/SW/2013 | H3N2 | 0.039 | 0.155 | 0.094 | 0.045 |
| A/Victoria/361/2011 | A/VC/2011 | H3N2 | 0.082 | 0.175 | 0.091 | 0.039 |
| A/Texas/12/2007, NA:E119V | A/TX/2007 E119V | H3N2 | 0.910 | 0.445 | 0.299 | 0.398 |
| A/HongKong/1968 | A/HK/1968 | H3N2 | 0.056 | 0.104 | 0.083 | 0.023 |
| A/Darwin/6/2021 | A/DW/2021 | H3N2 | 1.128 | >50 | 2.790 | >50 |
| A/Michigan/173/2020 | A/MC/2020 | H3N2 | 3.093 | 33.080 | 1.772 | 6.195 |
| A/Cambodia/E0826360/2020 | A/CB/2020 | H3N2 | 1.614 | 24.300 | 1.474 | 18.578 |
| A/Tasmania/503/2020 | A/TS/2020 | H3N2 | 2.007 | 5.066 | 1.181 | 26.131 |
| A/SouthAustralia/34/2019 | A/SA/2019 | H3N2 | 0.760 | 2.977 | 1.108 | 4.402 |
| A/HongKong/2671/2019 | A/HK/2019 | H3N2 | 0.073 | 5.068 | 0.190 | 21.468 |
| A/Kansas/14/2017 | A/KS/2017 | H3N2 | 0.284 | 0.485 | 0.559 | 4.522 |
| A/Switzerland/8060/2017 | A/SW/2017 | H3N2 | 0.063 | 0.029 | 0.031 | 0.342 |
| A/Singapore/INFIMH-16-0019/2016 | A/SG/2016 | H3N2 | 0.162 | >50 | 0.916 | >50 |
| B/Austria/1359417/2021 | B/AS/2021 | Victoria | 0.032 | 0.030 | 0.031 | 4.331 |
| B/Washington/02/2019 | B/WA/2019 | Victoria | 0.031 | 0.019 | 0.063 | 5.605 |
| B/Brisbane/35/2018 | B/BR/2018 | Victoria | 0.026 | 0.024 | 0.020 | >50 |
| B/North Carolina/25/2018 | B/NC/2018 | Victoria | 0.044 | 0.040 | 0.087 | 7.450 |
| B/Missouria/12/2018 NA:D197E | B/MS/2018 D197E | Victoria | 0.178 | 0.033 | 0.160 | 4.535 |
| B/Colorado/6/2017 | B/CL/2017 | Victoria | 0.669 | 0.042 | 1.171 | 1.632 |
| B/Laos/0654/2016, NA: 134N | B/LS/2016 134N | Victoria | 0.031 | 0.017 | 0.026 | 48.727 |
| B/Laos/0080/2016 | B/LS/2016 | Victoria | 0.047 | 0.018 | 0.029 | 13.520 |
| B/Brisbane/60/2008 | B/BR/2008 | Victoria | 1.161 | 0.191 | 0.152 | >50 |
| B/Utah/9/2014 | B/UT/2014 | Yamagata | 0.245 | 0.392 | 0.391 | >50 |
| B/Phuket/3073/2013 | B/PK/2013 | Yamagata | 0.193 | 0.117 | 0.222 | >50 |
| B/Massachusetts/2/2012 | B/MA/2012 | Yamagata | 0.204 | 0.125 | 0.472 | >50 |
| B/Texas/6/2011 | B/TX/2011 | Yamagata | 0.104 | 0.062 | 0.229 | >50 |
| B/Lee/1940 | B/Lee/1940 | Ancestral | 0.051 | 0.071 | 0.211 | 1.102 |
