# Supplementary material for: A pan-influenza antibody inhibiting neuraminidase via receptor mimicry
Source: Nature. 2023 May 31;618(7965):590–7. doi: 10.1038/s41586-023-06136-y (PMC10266979; doi:10.1038/s41586-023-06136-y)
Supplement: Supplementary file 5 — List of NA antigens used in the study and corresponding binding affinity values (KD) for FNI9, FNI17, FNI19 and 1G01 Fabs as measured by SPR. This table summarises the binding affinity measure by surface plasmon resonance (SPR) for FNI9, FNI17, FNI19, and 1G01 Fabs versus the N1 or N2 antigens described in the article. [file 41586_2023_6136_MOESM5_ESM.docx]

**Supplementary Table 4. List of NA antigens used in the study and corresponding binding affinity values (K_D_) for FNI9, FNI17, FNI19 and 1G01 Fabs as measured by SPR.**

| **NA antigen** | **aa mutation** | **N245 glycan** | **K_D_(nM)** | | | |
| --- | --- | --- | --- | --- | --- | --- |
|  |  |  | **1G01** | **FNI9** | **FNI17** | **FNI19** |
| N2 A/Hong Kong/2671/2019 | N245S | No | <0.02 | 3.49 | 122 | 3.71 |
| N2 A/Hong Kong/2671/2019 | N/A | Yes | 375 | 16.0 | 2095 | 15.1 |
| N2 A/Switzerland/8060/2017 | N245S | No | <0.02 | 4.76 | 353 | 4.42 |
| N2 A/Switzerland/8060/2017 | N/A | Yes | 398 | 17.1 | 3376 | 17.6 |
| N2 A/Tanzania/205/2010 | N/A | No | <0.02 | 4.08 | 27.3 | 0.92 |
| N2 A/Tanzania/205/2010 | S245N S247T | Yes | 194 | 5.00 | 234 | 8.55 |
| N2 A/Hong Kong/2671/2019 | K431E | Yes | ND | 44.24 | NB | ND |
| N1 A/Vietnam/1203/2004 | N/A | No | ND | 0.06 | 0.41 | ND |
| N1 A/Vietnam/1203/2004 | I203R (I223R) | No | ND | 882.05 | 100.01 | ND |
| N1 A/Vietnam/1203/2004 | R132I (R152I) | No | ND | NB | 2684 | ND |
| N1 A/Vietnam/1203/2004 | D179N (D199N) | No | ND | 17.44 | 37.71 | ND |
| N1 A/Vietnam/1203/2004 | S227R (S247R) | No | ND | 712.26 | 39.40 | ND |

Numbering in brackets corresponds to amino acids in N1 A/California/09/2009. Each K_D_ value is an average of at least 2 technical replicates. N/A: non-applicable. For measurements where the dissociation constant k_d_ is slower than the instrument’s detection range (k_d_ < 10^-6^ s^-1^), the binding affinity (K_D_) is denoted as <0.02 (nM). NQ: binding not quantifiable with a K_D_ value. NB: no binding. ND: not determined.
