# Supplementary material for: A pan-influenza antibody inhibiting neuraminidase via receptor mimicry
Source: Nature. 2023 May 31;618(7965):590–7. doi: 10.1038/s41586-023-06136-y (PMC10266979; doi:10.1038/s41586-023-06136-y)
Supplement: Supplementary file 6 — Cryo-EM data collection, refinement and validation statistics This table summarises the data collection, refinement and validation statistics for the Cryo-EM structures presented in the article. [file 41586_2023_6136_MOESM6_ESM.docx]

**Supplementary Table 5. Cryo-EM data collection, refinement and validation statistics**

|  | FNI9 (3-Fab):N2  A/Tanzania/  205/2010 (EMDB-29704)  (PDB 8G3M) | FNI9 (4-Fab):N2  A/Tanzania/  205/2010 (EMDB-29705)  (PDB 8G3N) | FNI9 (3-Fab): N2  A/Hong Kong/  2671/2019  (EMDB-29706)  (PDB 8G3O) | FNI9 (4-Fab): N2  A/Hong Kong/  2671/2019  (EMDB-29707)  (PDB 8G3P) | FNI17 (3-Fab): N2 A/Tanzania/  205/2010  (EMDB-29708)  (PDB 8G3Q) |
| --- | --- | --- | --- | --- | --- |
| **Data collection and processing** |  |  |  |  |  |
| Magnification | 81,000 | 81,000 | 105,000 | 105,000 | 105,000 |
| Voltage (kV) | 300 | 300 | 300 | 300 | 300 |
| Electron exposure (e–/Å^2^) | 48 | 48 | 52.32 | 52.32 | 52.61 |
| Defocus range (μm) | 1.6-3.2 | 1.6-3.2 | 1.2-1.8 | 1.2-1.8 | 1.3-2.0 |
| Pixel size (Å) | 1.06 | 1.093 | 0.832 | 0.832 | 0.82 |
| Symmetry imposed | C1 | C4 | C1 | C4 | C1 |
| Initial particle images (no.) | 1,014,489 | 1,014,489 | 466,868 | 466,868 | 1,852,675 |
| Final particle images (no.) | 217,552 | 177,658 | 58,739 | 162,364 | 651,664 |
| Map resolution (Å)  FSC threshold | 3.0  0.143 | 2.9  0.143 | 3.1  0.143 | 2.5  0.143 | 2.3  0.143 |
| Map resolution range (Å) | 3.0-157.4 | 2.9-160.0 | 3.1-180.9 | 2.5-176.0 | 2.3-144.3 |
|  |  |  |  |  |  |
| **Refinement** |  |  |  |  |  |
| Initial model used (PDB code) | none | none | none | none | none |
| Model resolution (Å)  FSC threshold | 3.2  0.5 | 2.9  0.5 | 3.3  0.5 | 2.7  0.5 | 2.5  0.5 |
| Model resolution range (Å) | 179.4-2.2 | 179.4-2.2 | 149.1-1.7 | 149.1-1.7 | 107.1-1.7 |
| Map sharpening *B* factor (Å^2^) | 44.1 | 44.7 | 53.7 | 71.8 | 66 |
| Model composition  Non-hydrogen atoms  Protein residues  Ligands | 17,510  2,369  1,157 | 19,344  2,593  977 | 17,056  2,353  981 | 19,340  2,593  981 | 17,468  2,362  1,150 |
| *B* factors (Å^2^)  Protein  Ligand | 99.8  193.4 | 91.7  149.9 | 89.3  189.8 | 77.4  128.6 | 60.8  130.7 |
| R.m.s. deviations  Bond lengths (Å)  Bond angles (°) | 0.0153  1.85 | 0.0154  1.82 | 0.0139  1.80 | 0.136  1.74 | 0.0130  1.85 |
| Validation  MolProbity score  Clashscore  Poor rotamers (%) | 0.89  0.14  0.21 | 0.88  0.08  0.0 | 0.92  0.31  0.0 | 0.94  0.38  0.0 | 1.01  0.91  0.21 |
| Ramachandran plot  Favored (%)  Allowed (%)  Disallowed (%) | 95.25  4.57  0.18 | 94.94  4.9  0.16 | 95.65  3.77  0.58 | 95.66  3.86  0.48 | 96.44  3.35  0.21 |

**Supplementary Table 5 (continued). Cryo-EM data collection, refinement and validation statistics**

|  | FNI17 (1-Fab): N2 A/Tanzania/  205/2010  S245N S247T (EMDB-29709)  (PDB 8G3R) | FNI19 (4-Fab):N2  A/Tanzania/  205/2010 (EMDB-29686)  (PDB 8G30) | FNI19 (3-Fab): N2  A/Hong Kong/  2671/2019  (EMDB-29712)  (PDB 8G40) | FNI19 (4-Fab): N2  A/Hong Kong/  2671/2019  (EMDB-29710)  (PDB 8G4V) | FNI17 (4-Fab): NA B/Massachusetts/  02 2012  (EMDB-29711)  (PDB 8G3Z) |
| --- | --- | --- | --- | --- | --- |
| **Data collection and processing** |  |  |  |  |  |
| Magnification | 105,000 | 150,000 | 105,000 | 105,000 | 105,000 |
| Voltage (kV) | 300 | 200 | 300 | 300 | 300 |
| Electron exposure (e–/Å^2^) | 53.81 | 19.35 | 53.84 | 53.84 | 52.61 |
| Defocus range (μm) | 1.2-1.5 | 1.5-2.5 | 1.2-1.5 | 1.2-1.5 | 1.0-2.0 |
| Pixel size (Å) | 0.834 | 0.927 | 0.834 | 0.834 | 0.82 |
| Symmetry imposed | C1 | C4 | C1 | C4 | C4 |
| Initial particle images (no.) | 2,326,510 | 874,485 | 1,940,956 | 1,940,956 | 240,515 |
| Final particle images (no.) | 528,681 | 170,901 | 121,274 | 885,566 | 177,249 |
| Map resolution (Å)  FSC threshold | 2.3  0.143 | 3.1  0.143 | 2.8  0.143 | 2.2  0.143 | 2.3  0.143 |
| Map resolution range (Å) | 2.3-180.9 | 3.1-174.3 | 2.8-177.6 | 2.2-141.1 | 2.3-178.8 |
|  |  |  |  |  |  |
| **Refinement** |  |  |  |  |  |
| Initial model used (PDB code) | none | none | none | none | none |
| Model resolution (Å)  FSC threshold | 2.5  0.5 | 3.1  0.5 | 2.8  0.5 | 2.2  0.5 | 2.4  0.5 |
| Model resolution range (Å) | 135.5-1.7 | 121.1-1.9 | 135.5-1.7 | 135.5-1.7 | 135.5-1.7 |
| Map sharpening *B* factor (Å^2^) | 62.5 | 143.3 | 55.5 | 64.0 | 65.8 |
| Model composition  Non-hydrogen atoms  Protein residues  Ligands | 15,369  2,081  1,063 | 19,354  2,594  1,095 | 17,497  2,360  1,153 | 19,328  2,561  717 | 19,845  2,525  117 |
| *B* factors (Å^2^)  Protein  Ligand | 87.1  120.5 | 117.5  222.3 | 61.1  176.2 | 57.3  87.4 | 59.6  109.9 |
| R.m.s. deviations  Bond lengths (Å)  Bond angles (°) | 0.0132  1.79 | 0.0147  1.96 | 0.0135  1.75 | 0.0131  1.75 | 0.0130  1.75 |
| Validation  MolProbity score  Clashscore  Poor rotamers (%) | 0.88  0.41  0.12 | 1.15  0.7  0.56 | 1.01  0.55  0.42 | 0.97  0.54  0.0 | 1.38  0.97  2.16 |
| Ramachandran plot  Favored (%)  Allowed (%)  Disallowed (%) | 96.42  3.38  0.2 | 93.46  6.22  0.32 | 95.32  4.37  0.31 | 95.8  3.88  0.32 | 95.1  4.7  0.2 |
